# Expression Profiles of Long Noncoding RNAs and Messenger RNAs in Mn-Exposed Hippocampal Neurons of Sprague–Dawley Rats Ascertained by Microarray: Implications for Mn-Induced Neurotoxicity

**DOI:** 10.1371/journal.pone.0145856

**Published:** 2016-01-08

**Authors:** Shuyan Ma, Li Qing, Xiaobo Yang, Guiqiang Liang, Li’e Zhang, Qin Li, Feng Xiong, Suwan Peng, Yifei Ma, Xiaowei Huang, Yunfeng Zou

**Affiliations:** 1 Teaching and Research Section of Hygienic Toxicology, School of Public Health, Guangxi Medical University, Nanning, Guangxi, People’s Republic of China; 2 Department of Occupational Health and Environmental Health, School of Public Health, Guangxi Medical University, Nanning, Guangxi, People’s Republic of China; Indian Institute of Integrative Medicine, INDIA

## Abstract

Manganese (Mn) is an essential trace element, while excessive expose may induce neurotoxicity. Recently, lncRNAs have been extensively studied and it has been confirmed that lncRNAs participate in neural functions and aberrantly expressed lncRNAs are involved in neurological diseases. However, the pathological effects of lncRNAs on Mn-induced neurotoxicity remain unclear. In this study, the expression profiles of lncRNAs and messenger RNAs (mRNAs) were identified in Mn-treated hippocampal neurons and control neurons via microarray. Bioinformatic methods and intersection analysis were also employed. Results indicated that 566, 1161, and 1474 lncRNAs meanwhile 1848, 3228, and 4022 mRNAs were aberrantly expressed in low, intermediate, and high Mn-exposed groups compared with the control group, respectively. Go analysis determined that differentially expressed mRNAs were targeted to biological processes, cellular components, and molecular functions. Pathway analysis indicated that these mRNAs were enriched in insulin secretion, cell cycle, and DNA replication. Intersection analysis denominated that 135 lncRNAs and 373 mRNAs were consistently up-regulated while 150 lncRNAs and 560 mRNAs were consistently down-regulated. Meanwhile, lncRNA BC079195 was significantly up-regulated while lncRNAs uc.229- and BC089928 were significantly down-regulated in three comparison groups. The relative expression levels of 3 lncRNAs and 4 mRNAs were validated through qRT-PCR. To the best of our knowledge, this study is the first to identify the expression patterns of lncRNAs and mRNAs in hippocampal neurons of Sprague–Dawley rats. The results may provide evidence on underlying mechanisms of Mn-induced neurotoxicity, and aberrantly expressed lncRNAs/mRNAs may be useful in further investigations to detect early symptoms of Mn-induced neuropsychiatric disorders in the central nervous system.

## Introduction

Mn is a common and ubiquitous metal in nature, accounting for nearly 0.1% of the Earth’s crust [1]. Human exposure to Mn through various routes (e.g., food, water, air, peripheral environment, and occupational workplace) is practically inevitable. As an essential trace element for human beings and other animals, Mn serves important functions in normal physiological processes, such as proper bone formation and brain development, reproduction, immune function, the metabolism of proteins, lipids, amino acids and carbohydrates, as well as in defence against oxidative stress [2,3]. Meanwhile, Mn as a cofactor interacts with multiple enzymes and exerts vital functions in biological organisms [4]. It is widely known that Mn deficiency rarely occurs in human beings because of the abundance of Mn in the diet. However, excessive exposure to Mn may produce neurotoxic effects. The brain is the target organ of Mn, such that large amounts of Mn accumulating in the central nervous system, especially in the basal ganglia, lead to certain neurological dysfunctions known as manganism, which has features similar to Parkinsonism [5]. Cases of manganism have been reported in a previous study on occupational workers [6]. Recent advances in technology and economic development, as well as improvements in protective awareness, helped reduce the occurrence of manganism. However, long-term chronic exposure still exists and leads to Mn neurotoxicity, early symptoms of which appear in the central nervous system. These symptoms include motor dysfunction, impaired working memory, oral expression disability, cognitive deficits, and damaged visuospatial function [7,8]. Moreover, a number of studies determined that the hippocampus is a pivotal structure associated with learning and memory ability; excessive Mn deposition in the hippocampus may induce cognitive dysfunction [9]. Our previous study investigated workers in a ferromanganese refinery company and revealed that occupational Mn exposure results in varying degrees of cognitive impairment [10]. In addition, high Mn exposure has also been implicated in several neurodegenerative disorders, such as Alzheimer’s disease (AD), Parkinson’s disease (PD), and Huntington’s disease (HD) that were reported in early research [11–15]. Although numerous efforts have been exerted to verify the potential mechanisms of Mn-induced neurotoxicity, the underlying molecular mechanisms remain elusive.

Long noncoding RNAs (lncRNAs) are currently defined as a kind of transcript without protein-coding capability. These molecules are longer than 200 nucleotides and are widespread in the cytoplasm and nuclei of eukaryotes [16]. lncRNAs are categorized as intronic, intergenic, sense, antisense, and bidirectional [17]. In the past, lncRNAs were once considered “transcriptional noise”. In recent years, lncRNAs have been extensively studied and a flurry of evidence demonstrated that the vital regulatory roles of lncRNAs in many biological processes, such as regulation of DNA metabolism, X chromosome inactivation, transcription activation, and chromatin modification [18–20]. In addition, lncRNAs have been functionally associated with many human diseases, such as cancer (e.g., hepatocellular carcinoma, lung adenocarcinoma, and colorectal cancer) [21–23] and heart failure [17]. Meanwhile, research has shown that lncRNAs are related to the regulation of brain development, neural plasticity, and cognitive function [24]. The aberrant expression of lncRNAs has been implicated in the pathogenesis of several neurodegenerative diseases, including HD, AD, and PD [25–27]. However, insufficient information is available about the effect of Mn on lncRNAs in the context of neurological deficits and about the role of lncRNAs in Mn-induced neurological disorders.

In the present study, we examined the expression profiles of lncRNAs and mRNAs in hippocampal neurons of Sprague–Dawley (SD) rats via microarray to identify aberrantly expressed lncRNAs and mRNAs. The neurons were exposed to different doses of Mn, whereas untreated normal cells served as a negative control. Subsequently, gene ontology (GO) analysis and pathway analysis were performed to predict the functions and possible pathways of differentially expressed mRNAs. Moreover, intersection analysis was conducted to select consistently up-regulated and down-regulated lncRNAs and mRNAs in the Mn-treated groups compared with the control group. Microarray results were verified via quantitative reverse transcription-polymerase chain reaction (qRT-PCR). To the best of our knowledge, this study is the first to analyze the lncRNAs and mRNAs of Mn-treated hippocampal neurons from SD rats. Our data may provide insights into physiology of lncRNAs/mRNAs and suggest novel mechanisms involved in Mn-induced neurological dysfunction.

## Materials and Methods

### Animals

Seventy-two healthy SD rats were obtained within 24 h of birth from the laboratory animal center of Guangxi Medical University (Guangxi, China; animal code SCXK 2009–0002). The Guangxi Medical University Animal Care and Use Committee approved all of the animal work. All experiments were performed in accord with the “Regulations for Studies with Experimental Animals”.

### Chemicals

MnCl_2_•4H_2_O, L-polylysine, D-Hank’s solution, and penicillin–streptomycin mixture were purchased from Sigma. DMEM-F12 medium was from HyClone. Neurobasal-A medium (without phenol red and serum) and fetal bovine serum (FBS) were purchased from Gibco. Human leukocyte antigen B27 (B27) was obtained from Invitrogen Life Technologies.

### Primary culture of SD rat hippocampal neurons

Rat hippocampal neurons were cultured as described by Li [28] with minor modifications. In brief, SD rats were sprayed with 75% ethanol within 24 h of birth. Brain tissues were obtained through decapitation. The hippocampal tissues were then rapidly stripped and placed into sterile precooled D-Hank’s solution. The hippocampal tissues were cut into small pieces and gently pipetted three times. The supernatant was collected and filtered through a 75 μm filter to prepare a cell suspension. The prepared cell suspension was centrifuged at 1000 rpm for 5 min. After centrifugation, the supernatant was discarded, and the pellet comprising hippocampal neurons was collected. The pelleted cells were diluted to a concentration of 1 × 10^6^ cells/mL using DMEM-F12 medium supplemented with 10% FBS, 1% penicillin–streptomycin mixture, and 1% B27 and then inoculated into flasks. To enhance cell attachment, each flask was pre-coated with 3 mL of L-polylysine (0.1 mg/mL) and incubated overnight at 37°C. After 24 h, the flasks were washed three times with D-Hank’s solution. The cells were incubated at 37°C in 5% CO_2_ for 24 h and then refreshed with serum-free neurobasal-A medium supplemented with 1% penicillin–streptomycin mixture and 1% B27. The cells were cultured for 8 d, and the culture medium was half refreshed every 3 d.

### Mn Exposure

We designed four treatment groups, including a control group and three groups treated with different Mn doses. This design was based on the results of previous experiments on cell viability (S1 Table and S1 Fig), neuronal morphology (S2 Fig), and apoptosis rate of Mn-exposed neurons in different groups (S2 Table). In specific, 8-day-old primary hippocampal neurons were exposed to 100, 400, and 800 μM MnCl_2_ for 24 h and designated as the low Mn-exposed (L), intermediate Mn-exposed (M), and high Mn-exposed (H) groups, respectively. The remaining neurons cultured for 24 h without Mn exposure were regarded as the control group (C). After 24 h of Mn exposure, the neurons were collected for follow-up experiments.

Before the follow-up experiments, in order to harvest enough neurons for further analysis, we first stripped and collected the hippocampus from six rats to prepare an initial cell suspension. Subsequently, the cells were cultured and treated with/without Mn, then used as one sample. Each group (including one negative control and three different Mn-exposed groups) contained three samples as biological replicate.

### RNA Extraction and RNA Quantity Control

Cellular total RNA was extracted from hippocampal neurons of 12 SD rat samples by using TRIzol (Invitrogen life technologies, Carlsbad, CA, USA) in accordance with the manufacturer’s protocol. The integrity of the RNA was assessed through standard denaturing agarose gel electrophoresis. A NanoDrop ND-1000 spectrophotometer was used to determine the quantity and quality of the total RNA.

### Microarray analysis of lncRNA and mRNA expression

An Agilent Array platform was employed to design the microarray. Sample preparation and microarray hybridization were performed in accordance with the manufacturer’s standard protocols with minor modifications. In brief, cellular mRNA was purified from the total RNA after the removal of rRNA (mRNA-ONLY Eukaryotic mRNA Isolation Kit, Epicentre). Subsequently, instead of pooling the mRNA, each sample was amplified and transcribed into fluorescent cRNA by using Agilent’s Quick Amp Labeling protocol (version 5.7, Agilent Technologies). The labeled cRNAs of 12 samples were individually hybridized onto the Rat lncRNA Array v1.0 (4 × 44K, Arraystar), which was designed for 9300 lncRNAs and 15200 protein coding transcripts. The lncRNAs were selected from the most authoritative databases of NCBI RefSeq and UCSC containing all mRNA records and orthologs of rat lncRNAs. Each transcript is accurately identified by a specific exon or splice junction probe, and the probe length is 60 mer. The information of microarray discussed in our manuscript have been deposited in National Center for Biotechnology Information (NCBI) Gene Expression Omnibus (GEO) and can be accessed through (GEO) Series accession number GPL15690 (http://www.ncbi.nlm.nih.gov/geo/query/acc.cgi?acc=GPL15690). After washing the slides, the arrays were scanned using Agilent Scanner G2505C.

Agilent Feature Extraction software (version 11.0.1.1) was used to analyze the acquired array images. Quantile normalization of the raw data and subsequent data processing were performed using GeneSpring GX v12.0 software (Agilent Technologies). After quantile normalization of the raw data, lncRNAs and mRNAs that flagged Present or Marginal (“All Targets Value”) in at least 3 out of 12 hippocampal neuron samples were chosen for further data analysis. Hierarchical clustering was performed to show distinguishable lncRNA and mRNA expression patterns among the hippocampal neuron samples. Differentially expressed lncRNAs/mRNAs with statistical significance between two groups were identified through scatter plot and volcano plot filtering with a threshold of fold-change ≥ 2 and p ≤ 0.05. Finally, GO and pathway analyses were applied to determine GO terms or the functions of these aberrantly expressed mRNAs in several biological pathways. Intersection analysis was used to screen consistently up-regulated and down-regulated lncRNAs and mRNAs in Mn-exposed groups compared with the control group. Microarray analysis was performed by Kang Chen Bio-Tech (Shanghai, People’s Republic of China).

### qRT-PCR validation assay

Total RNA was reverse-transcribed into cDNA by using SuperScript III Reverse Transcriptase (Invitrogen) for mRNAs and MMLV Reverse Transcriptase (Epicentre) for lncRNAs in accordance with the manufacturers’ instructions. Real-time PCR was performed on an Applied Biosystems ViiA 7 Real-time PCR System using a 2X PCR master mix (Arraystar). The specific primers for each gene are listed in S3 and S4 Tables. All reactions were performed in triplicate and normalized by the internal control product GAPDH. The median in each triplicate was used to calculate the relative levels of lncRNAs/mRNAs (DCt = Ct median of lncRNAs/mRNAs − Ct median of GAPDH). The data were expressed as fold changes (exposure vs. control).

### Statistical methods

Data are shown as mean ± standard deviation. Statistical analysis was performed for comparisons between two groups, whereas ANOVA was performed for multiple comparisons using Student’s t-test. The false discovery rate (FDR) was calculated to correct the p-value. Differences with p ≤ 0.05 were considered statistically significant between two groups. The fold changes and Student’s t-test were used to analyze the statistical significance of the microarray results. Fold change ≥ 2 and p ≤ 0.05 were considered as the threshold values to designate differentially expressed lncRNAs and mRNAs.

## Results

### Overview of the expression profiles of lncRNAs and mRNAs in hippocampal neurons of SD rats

**Fig 1:**
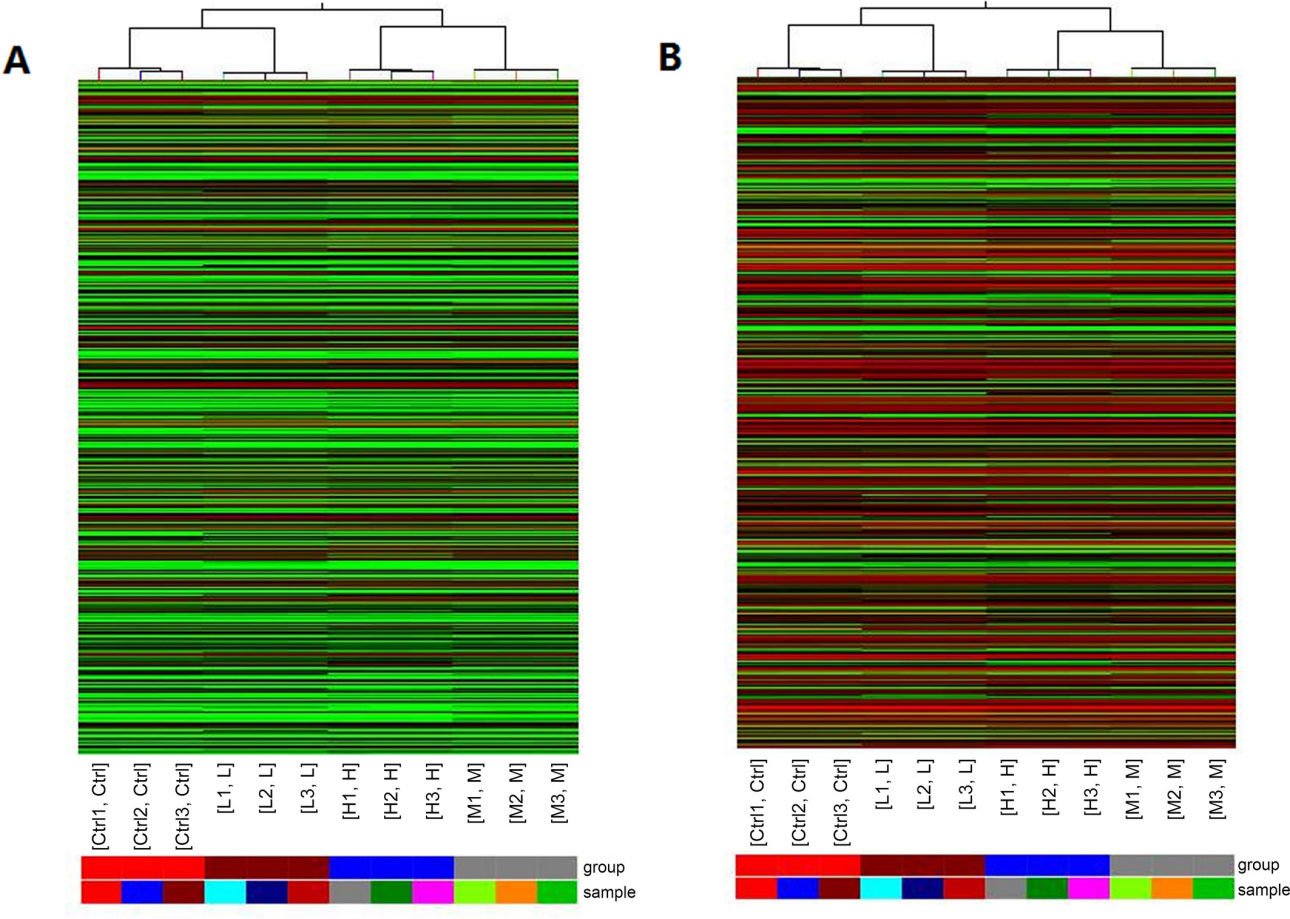
Heat map and hierarchical clustering of (A) lncRNA and (B) mRNA differential expression profiles between Mn-treated and control groups in 12 hippocampal neurons samples. Hierarchical clustering is a simple and commonly used clustering technique to analyze gene expression data. Cluster analysis arranges samples into groups on the basis of their expression levels, allowing us to hypothesize the relationships among samples. The dendrogram shows the relationships among the expression levels of samples. “Red” indicates high relative expression, whereas “Green” indicates low relative expression. Ctrl: untreated control group. L: low Mn-exposed group. M: intermediate Mn-exposed group. H: high-Mn-exposed group.

**Fig 2:**
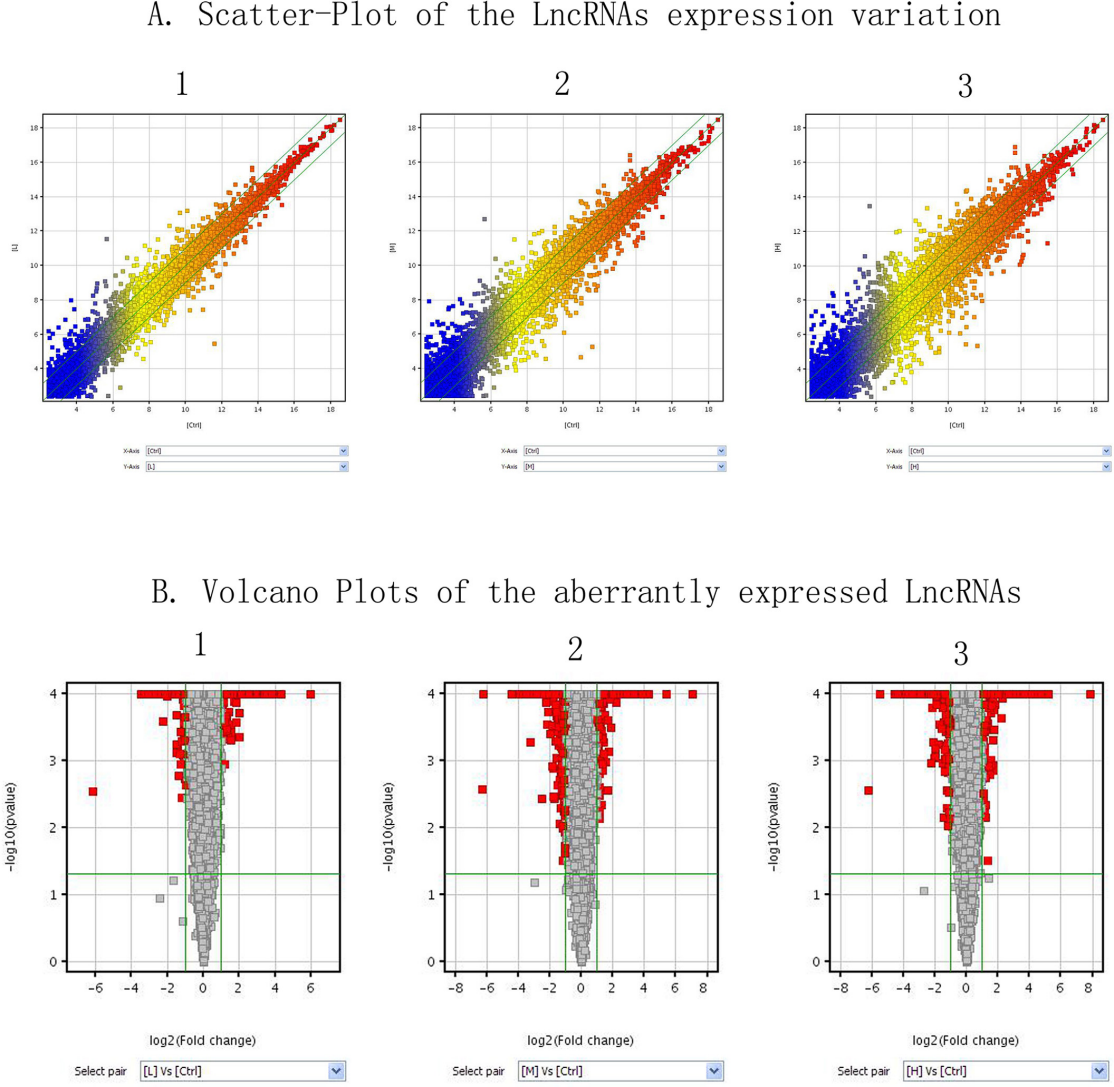
Overview of the microarray signatures. (A) Scatter plots showing the variations in lncRNA expression between two groups (1, L/C; 2, M/C; 3, H/C). A scatter plot is a visualization method used to assess the variation (or reproducibility) between chips. The values of the X and Y axes are the averaged normalized signal values of groups of samples (X: log2 scaled; and Y: −log10 scaled). The green lines are fold change lines (FC = 2). The color of the points indicates the intensities from low (blue) to high (red). The lncRNAs above the top green line and below the bottom green line indicate 2 FC between two groups of samples. (B) Volcano plots of the differentially expressed lncRNAs (1, L/C; 2, M/C; 3, H/C). A volcano plot is a useful tool for visualizing differential expression between two different conditions. The vertical lines correspond to twofold up and down, and the horizontal line represents a p-value of 0.05. The red point in the plot represents the differentially expressed lncRNAs with statistical significance.

**Fig 3:**
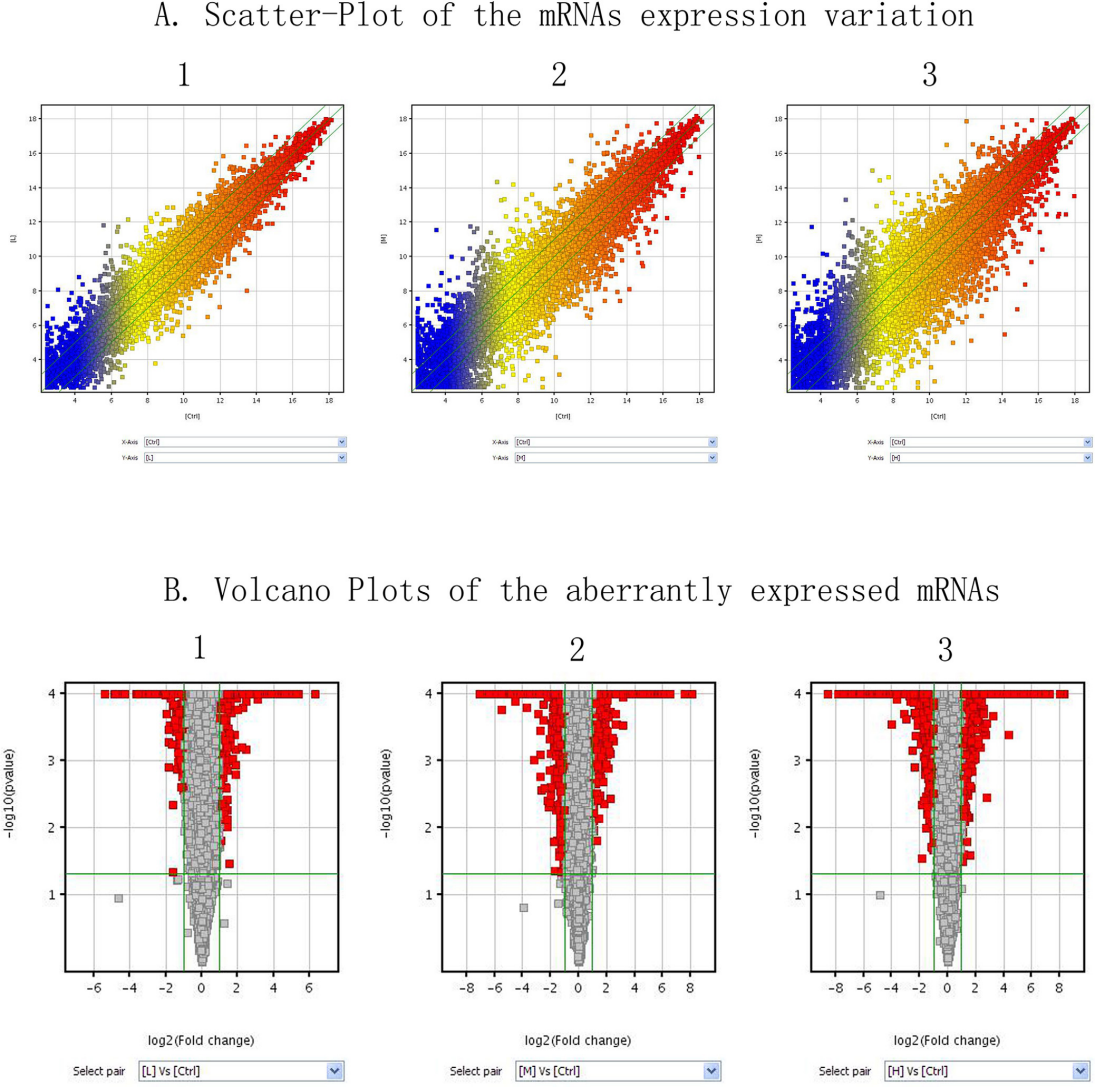
Overview of the microarray signatures. (A) Scatter plots showing the variations in the mRNA expression between two groups (1, L/C; 2, M/C; 3, H/C). A scatter plot is a visualization method used to assess the variation (or reproducibility) between chips. The values of the X and Y axes are the averaged normalized signal values of groups of samples (X: log2 scaled; and Y: −log10 scaled). The green lines are fold change lines (FC = 2). The color of the points indicates the intensities from low (blue) to high (red). The mRNAs above the top green line and below the bottom green line indicate 2 FC between two groups of samples. (B) Volcano plots of the differentially expressed mRNAs (1, L/C; 2, M/C; 3, H/C). A volcano plot is a useful tool for visualizing differential expression between two different conditions. The vertical lines correspond to twofold up and down, and the horizontal line represents a p-value of 0.05. The red point in the plot represents the differentially expressed mRNAs with statistical significance.

**Fig 4:**
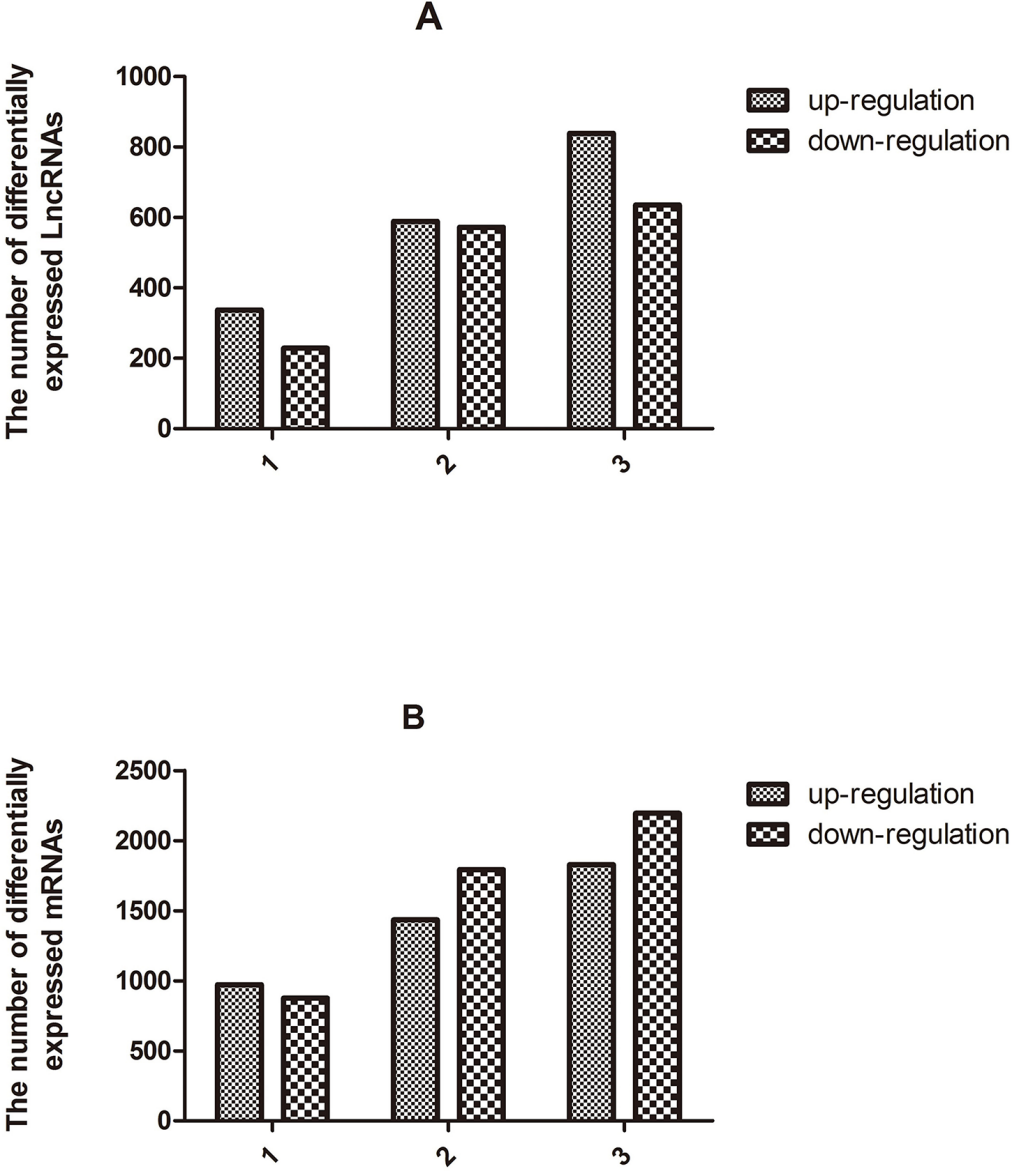
Number of differentially expressed lncRNAs (A) and mRNAs (B) between different groups (1, L/C; 2, M/C; 3, H/C).

**Fig 5:**
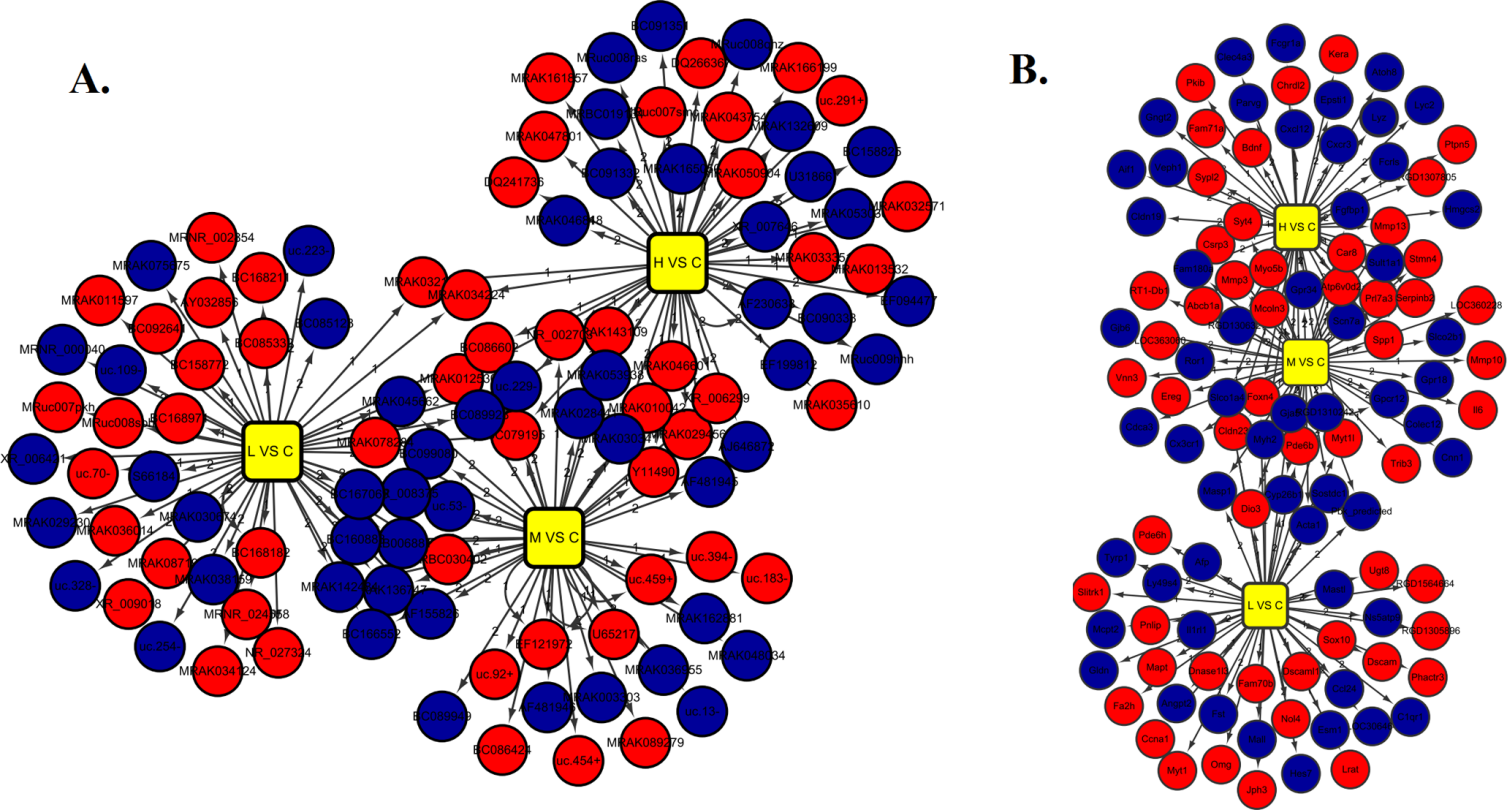
Network model analysis of the top 50 differentially expressed genes between three comparison groups. “Red” indicates high relative expression, whereas “Blue” indicates low relative expression.

To explore the potential biological functions of lncRNAs and mRNAs in Mn-induced neurotoxic effects, we examined the expression patterns of lncRNAs and mRNAs in the control and treatment groups. Organization of the expression profiles into heat maps showed the expression patterns of lncRNAs and mRNAs (Fig 1). To analyze the expression changes in lncRNAs and mRNAs among different groups, we classified these samples into low Mn-exposed vs. the control group (L/C), intermediate Mn-exposed vs. the control group (M/C), and high Mn-exposed vs. the control group (H/C). In total, we detected 7868 lncRNAs and 13836 mRNAs. Microarray results showed 566 (337 up-regulated and 229 down-regulated), 1161 (589 up-regulated and 572 down-regulated), and 1474 (839 up-regulated and 635 down-regulated) differentially expressed lncRNAs (fold change ≥ 2.0; p ≤ 0.05) in the L/C, M/C, and H/C groups, respectively. Meanwhile, 1848 (972 up-regulated and 876 down-regulated), 3228 (1435 up-regulated and 1793 down-regulated), and 4022 (1828 up-regulated and 2194 down-regulated) differentially expressed mRNAs (fold change ≥2.0; p ≤ 0.05) were detected in the L/C, M/C, and H/C groups, respectively. These results are shown through scatter plot and volcano plot filtering in Figs 2, 3 and 4, respectively. On the basis of our selection criteria (fold change ≥2.0; p ≤ 0.05), the top ten up-regulated and down-regulated lncRNAs and mRNAs in each pair of comparison groups are listed in Tables 1, 2, 3, 4, 5, 6, 7, 8, 9, 10, 11 and 12. In order to better describe the genetic changes between each group, we have drawn a figure of network model analysis based on the top fifty aberrantly expressed genes, shown in Fig 5.

**Table 1:** A collection of the top 10 up-regulated lncRNAs from the microarray data in L/C compare group.

L/C Up-regulated				
lncRNAs	Fold change	FDR	Regulation	Chromosome
BC079195	59.964523	3.54852E-07	up	chr15
MRBC030402	19.960125	3.50178E-07	up	chr15
BC158772	14.484746	1.16403E-06	up	chr16
uc.70-	13.564375	5.71553E-07	up	chr3
MRAK087199	12.8869	3.51631E-07	up	chr3
XR_009018	12.382811	2.38365E-07	up	chr7
BC086602	12.049046	2.5049E-07	up	chr12
MRAK036014	11.523903	1.02563E-07	up	chr5
BC092641	11.28802	5.74912E-06	up	chr3
AY032856	10.992081	1.61485E-07	up	chr16

**Table 2:** A collection of the top 10 down-regulated lncRNAs from the microarray data in L/C compare group.

L/C Down-regulated				
lncRNAs	Fold change	FDR	Regulation	Chromosome
uc.229-	70.1205	0.004736144	down	chr4
BC089928	11.344296	9.8427E-08	down	chr13
XR_006421	11.247746	5.31191E-05	down	chr5
uc.53-	9.882389	1.05187E-06	down	chr14
MRAK136747	8.467059	4.26495E-06	down	chr9
BC160888	6.9719667	1.02563E-07	down	chr5
MRNR_000040	6.288158	1.02563E-07	down	chr9
MRAK045662	5.6249037	2.17096E-06	down	chr1
uc.328-	5.224561	6.26487E-05	down	chr3
BC166552	5.211486	7.17231E-08	down	chr1

**Table 3:** A collection of the top 10 up-regulated lncRNAs from the microarray data in M/C compare group.

M/C Up-regulated				
lncRNAs	Fold change	FDR	Regulation	Chromosome
BC079195	132.77821	1.28881E-07	up	chr15
uc.394-	40.98603	7.40822E-07	up	chr1
MRAK012530	18.9673	3.28717E-08	up	chr16
EF121972	17.51655	1.64771E-07	up	chr10
MRBC030402	14.821312	1.84093E-07	up	chr15
Y11490	14.297066	5.59805E-07	up	chr16
uc.459+	14.277521	1.75475E-05	up	chrX
U65217	13.656247	2.52839E-07	up	chr20
BC086424	13.371725	2.34572E-06	up	chr14
MRAK143109	13.266635	4.11101E-07	up	chr5

**Table 4:** A collection of the top 10 down-regulated lncRNAs from the microarray data in M/C compare group.

M/C Down-regulated				
lncRNAs	Fold change	FDR	Regulation	Chromosome
uc.229-	79.71025	0.003835159	down	chr4
BC089928	76.679565	2.79968E-07	down	chr13
AJ646872	21.493917	9.21139E-08	down	chr2
MRAK028441	14.979339	1.63709E-07	down	chr16
MRAK030341	13.04273	7.75768E-08	down	chr8
BC167069	12.182365	1.80818E-06	down	chr3
AB006881	11.206064	7.40822E-07	down	chr8
MRAK142484	10.8857565	1.34958E-07	down	chr18
AF481945	10.659393	4.03043E-07	down	chr4
MRAK045662	10.6144905	2.01822E-06	down	chr1

**Table 5:** A collection of the top 10 up-regulated lncRNAs from the microarray data in H/C compare group.

H/C Up-regulated				
lncRNAs	Fold change	FDR	Regulation	Chromosome
BC079195	230.79996	7.37999E-08	up	chr15
MRAK143109	36.185135	1.51509E-07	up	chr5
MRAK050904	29.185905	2.51353E-06	up	chr17
MRAK013532	28.75131	2.32718E-08	up	chr6
MRAK012530	27.037758	1.48568E-08	up	chr16
MRAK032113	26.38797	1.01904E-06	up	chr10
BC086602	25.950098	4.80713E-08	up	chr12
DQ241736	25.467655	1.48568E-08	up	chr9
Y11490	24.860514	2.44551E-07	up	chr16
MRAK166199	23.475506	1.50208E-08	up	chr17

**Table 6:** A collection of the top 10 down-regulated lncRNAs from the microarray data in H/C compare group.

H/C Down-regulated				
lncRNAs	Fold change	FDR	Regulation	Chromosome
uc.229-	74.15845	0.004103726	down	chr4
BC089928	45.122936	6.16904E-08	down	chr13
MRuc008ras	23.616882	9.37168E-08	down	chr2
MRBC019134	22.496077	1.09058E-07	down	chr8
MRuc009hnh	22.312845	3.31288E-07	down	chr1
MRuc008qhz	20.594889	2.54127E-08	down	chr2
MRAK165050	20.347012	5.36152E-07	down	chr12
BC091332	19.79687	1.47466E-07	down	chr12
U31866	18.502028	2.17795E-08	down	chr14
MRAK030341	17.914583	3.56335E-06	down	chr8

**Table 7:** A collection of the top 10 up-regulated mRNAs from the microarray data in L/C compare group.

L/C Up-regulated				
mRNAs	Fold change	FDR	Regulation	Chromosome
Foxn4	78.262634	2.71601E-07	up	chr12
Pnlip	41.453915	7.15972E-08	up	chr1
Pde6h	37.808514	3.254E-07	up	chr4
Fam70b	35.685772	2.40361E-07	up	chr16
Pde6b	33.48449	0.000109602	up	chr14
Fa2h	27.836397	3.69043E-06	up	chr19
Myt1l	25.476294	6.18415E-07	up	chr6
Lrat	24.396181	2.11885E-06	up	chr2
Dscaml1	23.20265	5.04098E-07	up	chr8
Ccna1	17.553026	2.08119E-08	up	chr2

**Table 8:** A collection of the top 10 down-regulated mRNAs from the microarray data in L/C compare group.

L/C Down-regulated				
mRNAs	Fold change	FDR	Regulation	Chromosome
Acta1	42.775806	6.05199E-08	down	chr19
Cyp26b1	29.177687	6.38531E-08	down	chr4
Il1rl1	27.717012	6.38531E-08	down	chr9
Tyrp1	23.189777	1.69414E-07	down	chr5
Gja5	19.818396	9.69425E-08	down	chr2
Mall	12.749295	1.096E-07	down	chr3
Slco1a4	12.644817	1.35495E-07	down	chr4
Afp	12.033638	7.91548E-05	down	chr14
Gldn	11.984988	1.66106E-07	down	chr8
Angpt2	11.849187	1.0268E-06	down	chr16

**Table 9:** A collection of the top 10 up-regulated mRNAs from the microarray data in M/C compare group.

M/C Up-regulated				
mRNAs	Fold change	FDR	Regulation	Chromosome
Mmp13	267.45764	2.06335E-06	up	chr8
Cldn23	185.0186	1.88668E-08	up	chr16
Abcb1a	89.73269	5.59689E-08	up	chr4
Serpinb2	86.31821	1.9277E-07	up	chr13
Mmp3	75.23152	1.24733E-08	up	chr8
Car8	70.78515	1.24733E-08	up	chr5
Mcoln3	60.20042	2.66618E-08	up	chr2
Foxn4	58.90062	1.07712E-07	up	chr12
Csrp3	50.947685	1.14919E-06	up	chr1
Vnn3	39.468803	1.52229E-06	up	chr1

**Table 10:** A collection of the top 10 down-regulated mRNAs from the microarray data in M/C compare group.

M/C Down-regulated				
mRNAs	Fold change	FDR	Regulation	Chromosome
Gja5	137.88045	7.21376E-05	down	chr2
Slco1a4	120.98872	1.35059E-08	down	chr4
Gpr34	85.06124	9.68555E-08	down	chrX
Myh2	72.092186	7.99336E-08	down	chr10
Fgfbp1	64.54877	1.05439E-08	down	chr14
Fam180a	59.885002	1.1617E-06	down	chr4
Gpr18	46.87135	0.000271259	down	chr15
Cyp26b1	45.286465	4.04566E-08	down	chr4
Acta1	45.243572	1.49908E-08	down	chr19
Sostdc1	45.09045	1.46599E-07	down	chr6

**Table 11:** A collection of the top 10 up-regulated mRNAs from the microarray data in H/C compare group.

H/C Up-regulated				
mRNAs	Fold change	FDR	Regulation	Chromosome
Mmp13	308.32635	1.60249E-06	up	chr8
Cldn23	295.244	1.32246E-08	up	chr16
Serpinb2	250.94687	9.20009E-08	up	chr13
Car8	150.02893	6.44157E-09	up	chr5
Abcb1a	121.758675	3.31885E-08	up	chr4
Prl7a3	100.71297	5.76524E-07	up	chr17
Mcoln3	85.6437	2.06966E-08	up	chr2
Kera	69.951614	1.12425E-06	up	chr7
Myt1l	63.64153	1.63188E-07	up	chr6
Foxn4	60.241604	7.67439E-08	up	chr12

**Table 12:** A collection of the top 10 down-regulated mRNAs from the microarray data in H/C compare group.

H/C Down-regulated				
mRNAs	Fold change	FDR	Regulation	Chromosome
Fgfbp1	385.92288	1.9383E-07	down	chr14
RGD1306327	229.82156	4.32092E-08	down	chr15
Gja5	177.80765	5.47686E-07	down	chr2
Hmgcs2	137.29005	8.02645E-08	down	chr2
Gpr34	118.59217	3.83499E-09	down	chrX
Scn7a	113.32316	9.36239E-09	down	chr3
Slco1a4	97.509155	9.8841E-06	down	chr4
Fam180a	87.05466	2.27263E-08	down	chr4
Atoh8	77.70722	1.43208E-08	down	chr4
Fcgr1a	76.10863	5.51763E-07	down	chr2

### GO analysis

To further predict the functions of mRNAs identified from Mn-exposed neurons, we performed GO analysis on the aberrantly expressed mRNAs. Up to 13836 coding transcripts were detected in the 12 samples of SD rat hippocampal neurons. Through 15200 coding transcripts probes, we found that 1848, 3228, and 4022 mRNAs were differentially expressed in the L/C, M/C, and H/C groups, respectively.

GO analysis is a functional analysis that associates differentially expressed mRNAs with GO categories. The GO categories were derived from the GO database (http://www.geneontology.org) and involved three domains (biological process, cellular component, and molecular function) of defined terms that describe gene product attributes. Fisher’s exact test was used to determine whether or not the overlap between the DE list and the GO annotation list is higher than expected. The p-value denotes the significance of GO term enrichment in the differentially expressed genes. GO terms were considered statistically significant at p ≤ 0.05.

**Fig 6:**
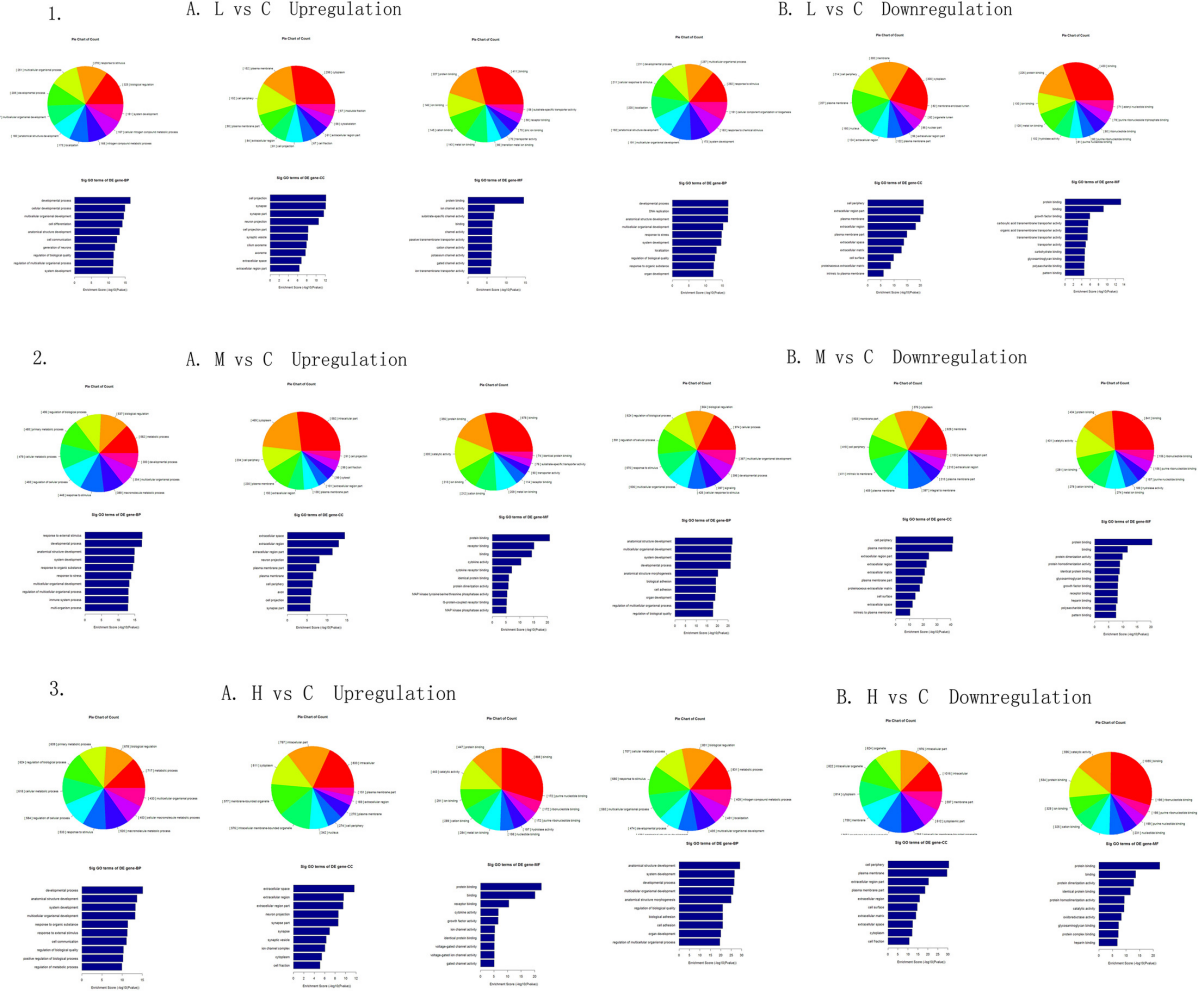
Gene ontology (GO) enrichment analysis for differentially expressed genes. GO analysis provides a controlled vocabulary to describe differentially expressed transcript attributes in all organisms. The ontology covers three domains: biological process (BP), cellular component (CC), and molecular function (MF). The p-value denotes the significance of GO term enrichment in the DE genes. GO terms are considered statistically significant at p ≤ 0.05. 1, L/C; 2, M/C; 3, H/C.

The top 10 genes related to GO term and the top 10 significant GO terms are shown in Fig 6. In the L/C group, the up-regulated mRNAs were involved in 795 biological processes, 73 cellular components, and 118 molecular functions. The down-regulated mRNAs were involved in 1010 biological processes, 101 cellular components, and 141 molecular functions. In the M/C group, the up-regulated mRNAs were involved in 1090 biological processes, 59 cellular components, and 163 molecular functions. The down-regulated mRNAs were involved in 1343 biological processes, 109 cellular components, and 195 molecular functions. In the H/C group, the up-regulated mRNAs were involved in 874 biological processes, 77 cellular components, and 168 molecular functions. The down-regulated mRNAs were involved in 1369 biological processes, 130 cellular components, and 188 molecular functions.

The highest enrichment scores in the biological process category appeared in the developmental process in the L/C group, the response to external stimulus and anatomical structure development in the M/C group, and the development process and anatomical structure development in the H/C group. In the cellular component category, the most significant terms were cell projection, synapse, and cell periphery in the L/C group, and extracellular space, cell periphery and plasma membrane in the M/C and H/C groups. In the molecular function category, the most represented GO term was protein binding in the L/C, M/C, and H/C groups (Fig 6).

### Pathway analysis

Basing from the latest Kyoto Encyclopedia of Genes and Genomes (http://www.genome.jp/kegg) database, we performed pathway enrichment analysis for aberrantly expressed protein coding genes. The p-value (Fisher p-value) denotes the significance of the pathway correlated to the conditions. The pathways were considered statistically significant at p ≤ 0.05.

**Fig 7:**
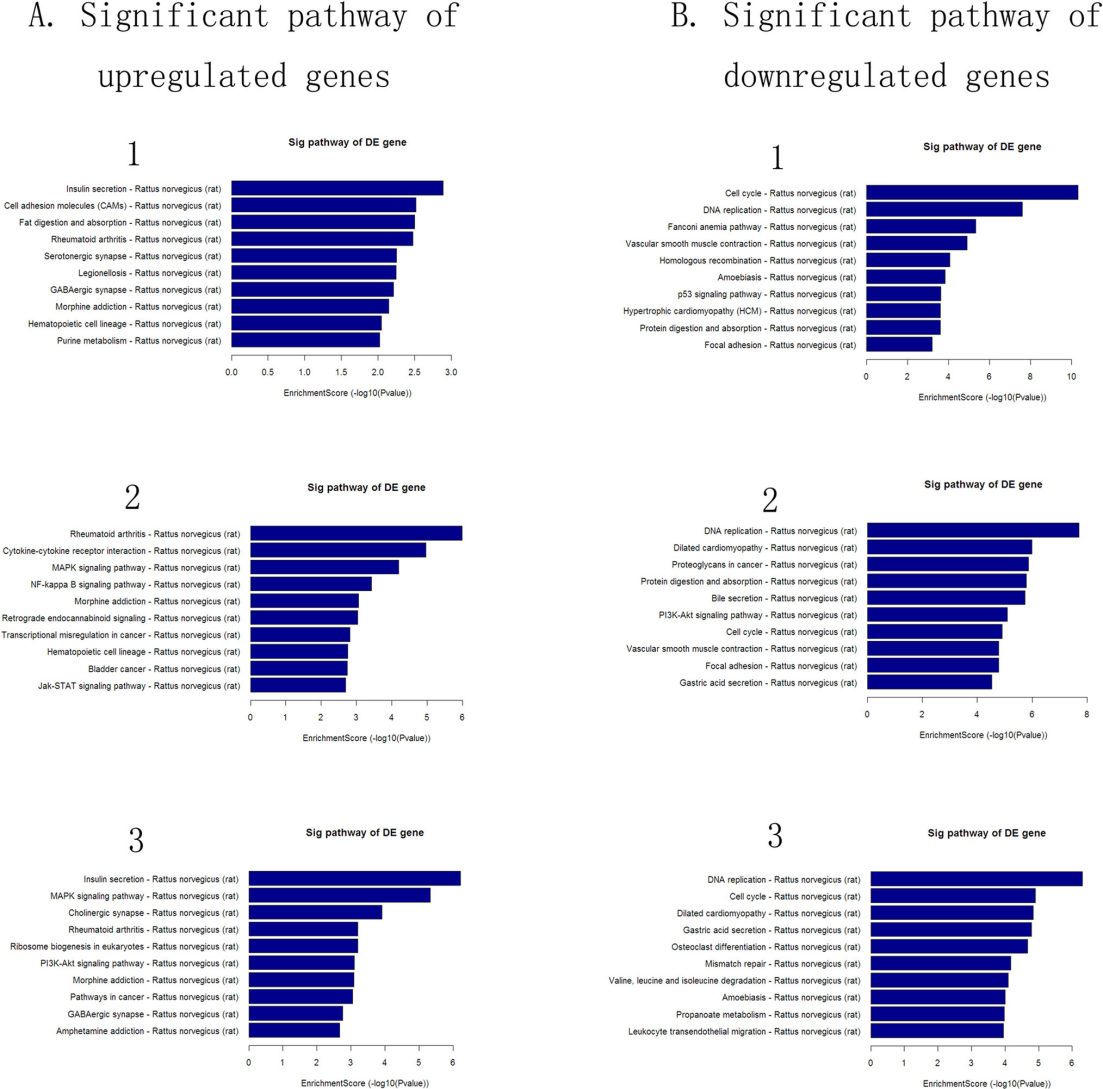
Pathway analysis of the differentially expressed genes. (A) Significant pathways of up-regulated genes. (B) Significant pathways of down-regulated genes. Pathway analysis is a functional analysis that maps genes to Kyoto Encyclopedia of Genes and Genomes pathways (http://www.genome.jp/kegg/). The p-value (Fisher p-value) denotes the significance of the pathway correlated with the conditions. The pathways are considered statistically significant at p < 0.05. 1, L/C; 2, M/C; 3, H/C.

The top 10 pathways of significant aberrantly expressed genes in the different groups are shown in Fig 7. The most enriched pathways in the pathway analysis were insulin secretion and cell cycle in the L/C group, rheumatoid arthritis and DNA replication in the M/C group, and insulin secretion and DNA replication in the H/C group.

### Intersection analysis

**Fig 8:**
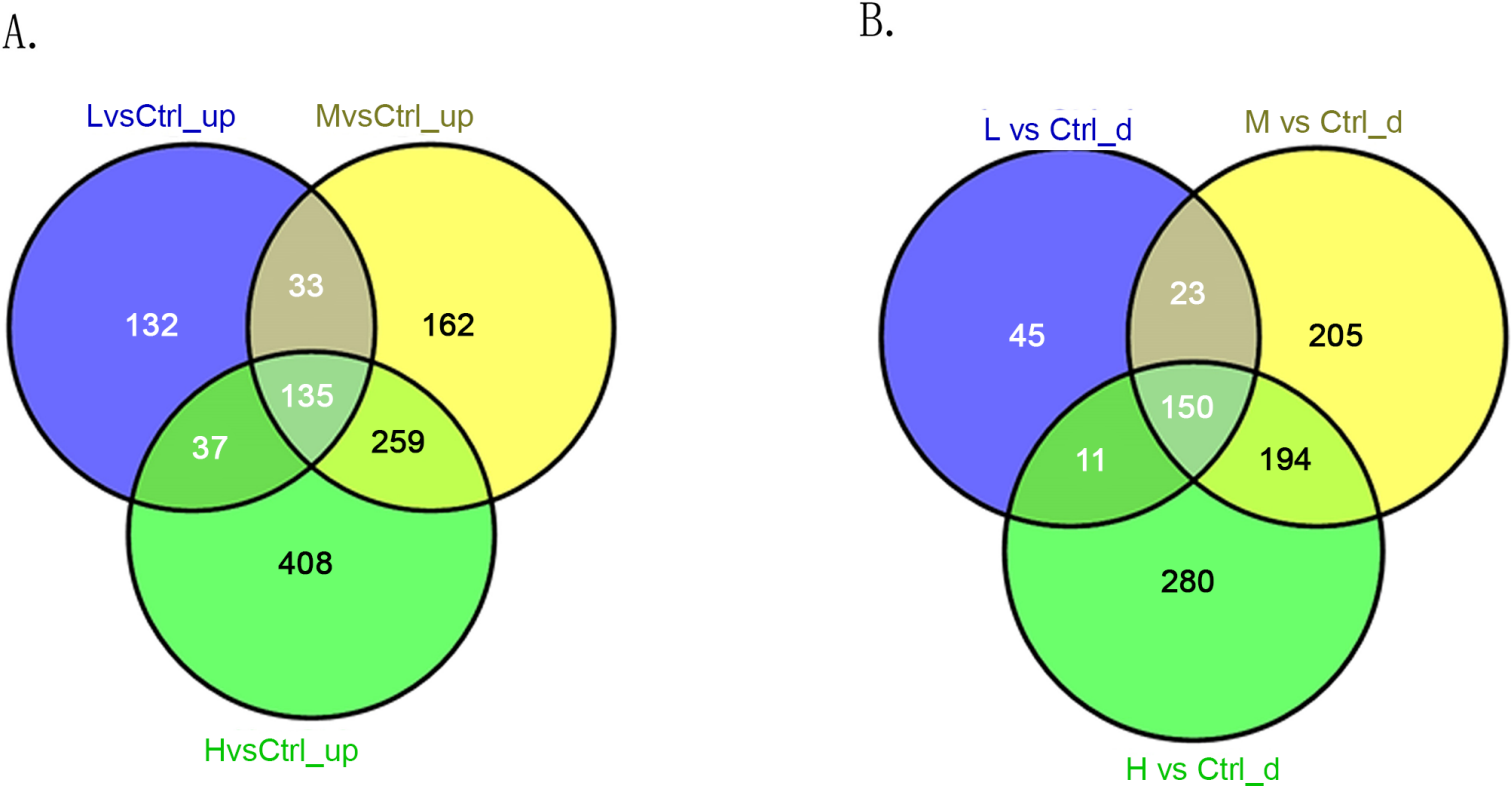
Intersection analysis of differentially expressed lncRNAs in three comparison groups (L/C, M/C, and H/C). (A) Consistently up-regulated lncRNAs. (B) Consistently down-regulated lncRNAs.

**Fig 9:**
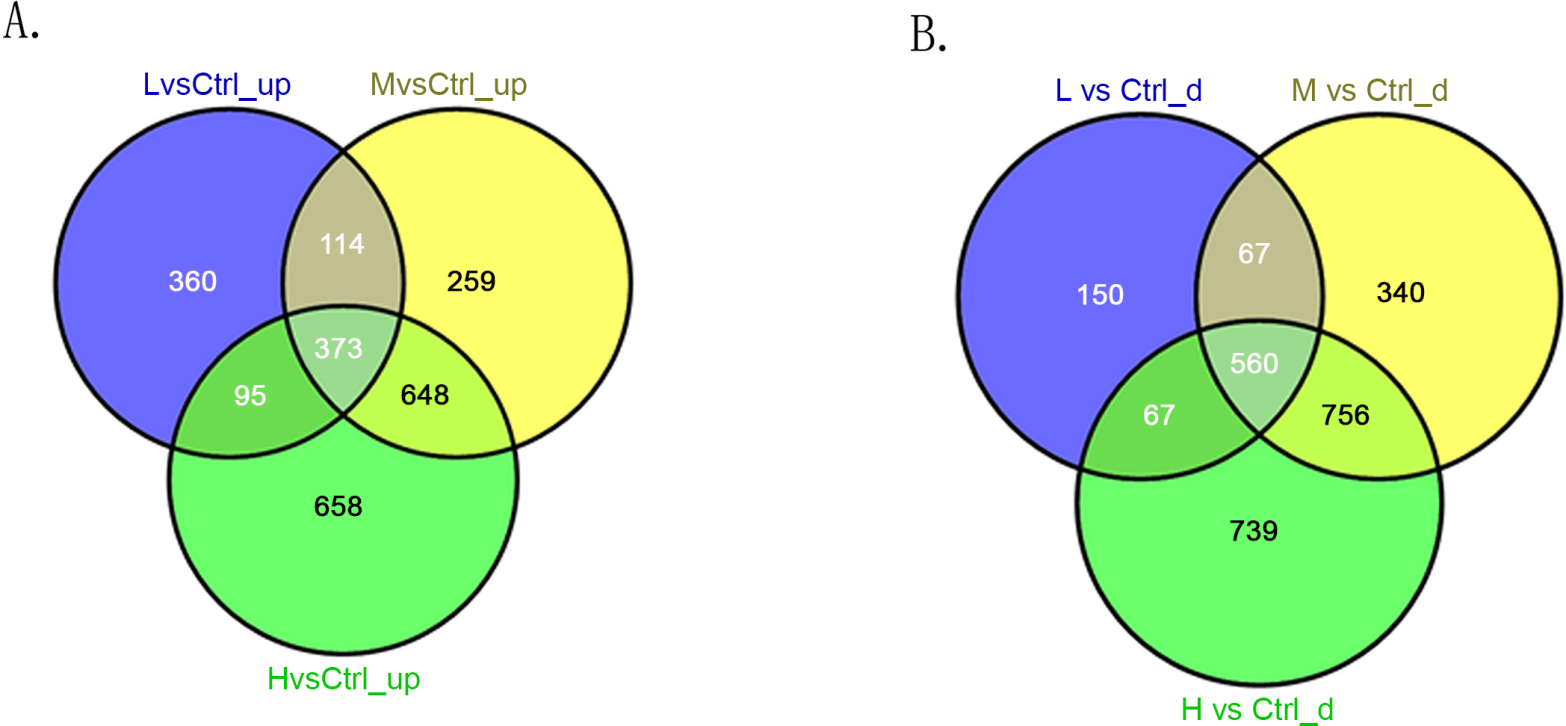
Intersection analysis of differentially expressed mRNAs in three comparison groups (L/C, M/C, and H/C). (A) Consistently up-regulated mRNAs. (B) Consistently down-regulated mRNAs.

In the three comparison groups (L/C, M/C, and H/C), we screened out lncRNAs and mRNAs with similar altered trends (consistently up-regulated or down-regulated) through intersection analysis. The consistently up-regulated and down-regulated lncRNAs in each comparison group are shown in Fig 8(A) and 8(B), respectively. The consistently up-regulated and down-regulated mRNAs in each comparison group are shown in Fig 9(A) and 9(B), respectively. As shown in Figs 8 and 9, 135 lncRNAs and 373 mRNAs were consistently up-regulated while 150 lncRNAs and 560 mRNAs were consistently down-regulated in the three comparison groups.

### Quantitative real-time PCR analysis of lncRNA and mRNA expression

**Fig 10:**
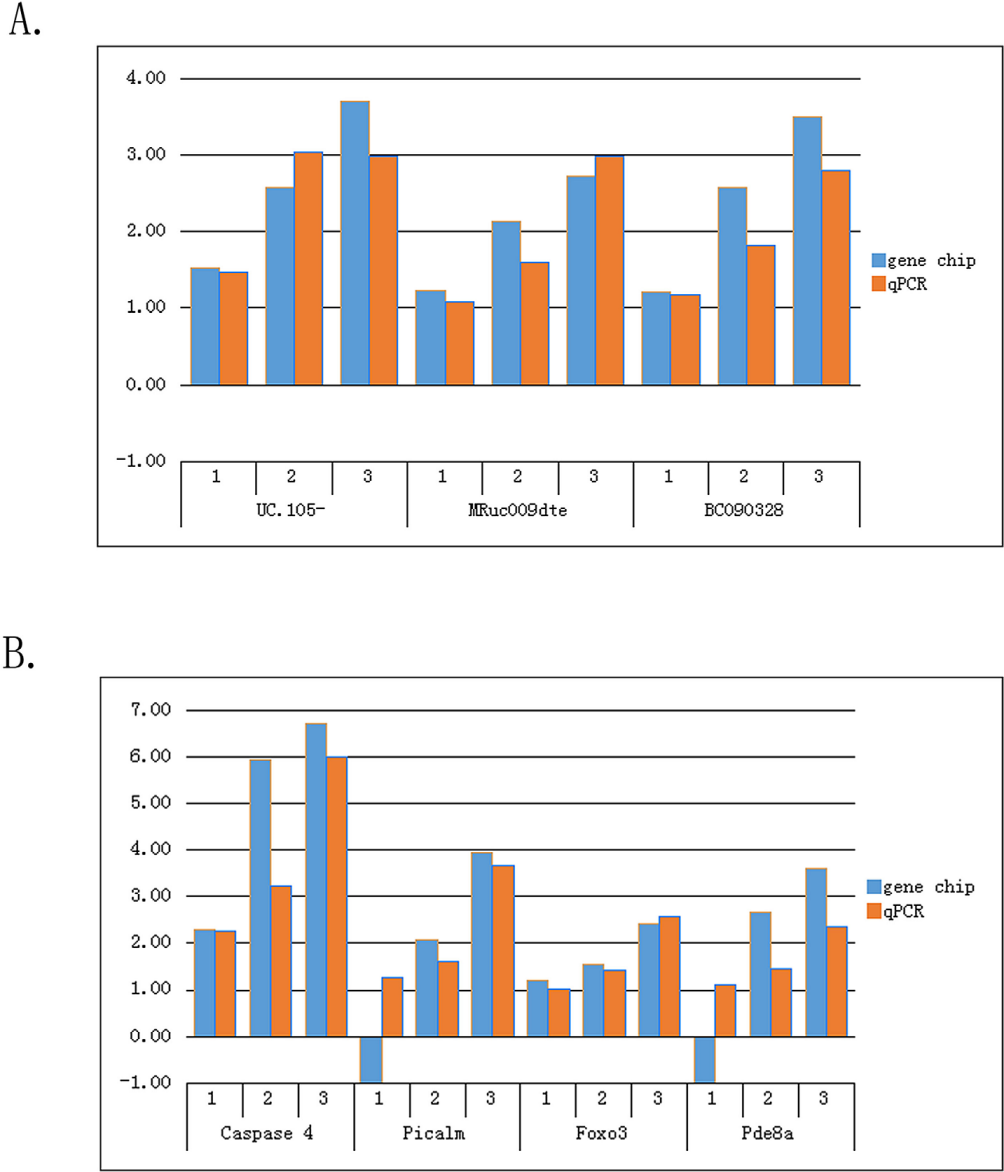
Comparison between gene chip data and qRT-PCR result. (A) Three lncRNAs (UC.105-, MRuc009dte, and BC090328) and (B) four mRNAs (Caspase 4, Picalm, Foxo3, and Pde8a) in 12 hippocampal neuron samples were validated through qRT-PCR. The heights of the columns in the chart represent fold changes. The validation results enunciate that the microarray data are consistent with the qRT-PCR results (1, L/C; 2, M/C; 3, H/C).

Quantitative real-time PCR was used to confirm the microarray data. We verified several lncRNAs and mRNAs of interest for further analysis. This verification was based on the characteristics of the differentially expressed lncRNAs, such as fold change, p-value, and the relationship among an lncRNA, its nearby coding gene, and the coordinate of the coding gene. We initially selected three interesting candidate lncRNAs (UC.105-, MRuc009dte, and BC090328). With respect to differentially expressed mRNAs, we primarily selected four related genes (Caspase 4, Picalm, Foxo3, and Pde8a), which we thought may play a crucial role in the onset and development of Mn-induced neurotoxicity. The results of qRT-PCR for the selected lncRNAs were consistent with the microarray data (Fig 10A). The expression levels of UC.105-, MRuc009dte, and BC090328 were significantly higher in the Mn-exposed groups than in the negative control group. The increase in expression level was dose dependent. Meanwhile, the expression of the selected mRNAs presented similar tendencies to their respective lncRNAs, except for Picalm and Pde8a in the L/C group, whose relative expression levels were in contrast with the results of the microarray (Fig 10B). This result can be considered a normal phenomenon because microarrays may sometimes generate false positive results.

## Discussion

As an essential trace element, Mn participates in the regulation of normal physiological processes. This element is mainly absorbed through the intestinal tract. In some environments containing high Mn levels, Mn may also be absorbed via inhalation. Subsequently, Mn is released into the blood. Excessive Mn is excreted via bile and feces. However, Mn can be transferred across the blood–cerebrospinal fluid (CSF) barrier and the blood–brain barrier [29]. Overexposure to this metal may lead to elevated levels of Mn in the brain and further induce neurotoxic effects [30]. Neurotoxicity caused by Mn exposure primarily occurs in certain occupational groups, such as miners, smelters, and welders [31–33]. Long-term exposure to high levels of airborne Mn in occupational settings may result in manganism, a serious and permanent neurodegenerative disorder that shares multiple similar characteristics with PD and was described for the first time by Couper in 1837 [34]. Cases of manganism have reduced in recent years. However, given its versatile chemical properties, Mn is widely used in industrial sites, such as for gasoline anti-knock additives (methylcyclopentadienyl manganese tricarbonyl), which may elevate the levels of Mn in the immediate environment [35,36]. Therefore, the initial neurotoxic effects of chronic Mn exposure represent a critical contemporary concern. Long-term chronic exposure to Mn may lead to the accumulation of this metal in the brain, potentially threatening the central nervous system. Moreover, as an important region of the brain, the hippocampus is crucial in learning, memory, and motivation [37]. Excessive Mn accumulation in this region may exert detrimental effects on cognitive function [38]. Previous studies found increased Mn levels in the plasma of AD patients and in the CSF of PD patients. Meanwhile, high Mn levels are a risk factor for AD and can induce Aβ-related cognitive dysfunction [39–41]. However, the mechanism underlying Mn-induced neurotoxicity remains unclear, and considerable effort must be exerted to obtain a comprehensive interpretation.

lncRNAs, transcripts that do not encode proteins, have been extensively studied in recent years. lncRNAs are not only involved in proper physiological functions but also related to the pathogenesis of many diseases [42]. Interestingly, the aberrant expression of lncRNAs is involved in the pathogenesis of certain neuropsychiatric disorders. Research has shown that lncRNAs are highly associated with brain development, synaptic plasticity, neural differentiation and maintenance, cognitive function, learning and memory ability, and neurodegenerative diseases [43]. In brain development, Pollard performed a genome comparison between human beings and chimpanzee. He found a specific expression of lncRNA HAR1F in the developing human neocortex from 7 to 19 gestational weeks, a pivotal period that is closely associated with the specification and migration of cortical neurons [44]. Regarding neural differentiation and maintenance, lncRNA AK053922 exerts a bifunctional role by inhibiting or activating sonic hedgehog signaling and further assisting in designating different types of neurons [45,46]. With respect to synaptic plasticity, Bernard indicated that metastasis-associated lung adenocarcinoma transcript 1 (Malat1) is highly expressed in cultured hippocampal neurons. Knockdown of Malat1 reduces synaptic density, whereas overexpression of this lncRNA leads to a cell-autonomous increase in synaptogenesis [47]. With regard to cognitive function and memory, dysfunction of anti-BDNF lncRNA (BDNF-AS, also denoted as BDNF-OS) by antagoNAT or siRNA up-regulates BDNF protein and further induces the growth and differentiation of neurons. This effect is in line with its role in learning and memory function [48,49]. With respect to neurodegenerative disorders, lncRNA17A participates in AD development by directly acting on GPR51 and further damaging GABAB receptor signaling [50]. Furthermore, lncRNAs SOX2OT, 1810014B01Rik, and BC200 are dysregulated in AD and PD, in which all of them are significantly up-regulated. In particular, SOX2OT lncRNA can regulate the development of the central nervous system and neurogenesis in adult mice, which may serve as a biomarker of neurodegeneration, including AD and PD [51,52].

In the present study, some of the differentially expressed mRNAs in Tables 7, 8, 9, 10, 11 and 12 have aroused our interest. These mRNAs include Foxn4, Myt1l, Fa2h, Dscaml1, and Hmgcs2. Foxn4 is a member of the forkhead/winged helix transcription factor family [53] that has attracted considerable attention because of its key roles in neurogenesis [54] and central nervous system lesion [55]. Chen established a spinal cord injury model in adult rats and indicated that Foxn4 is located on neurons and astrocytes. The expression of Foxn4 is higher in the model group than in the normal control group [56]. Our results were consistent with those of Chen’s. After Mn treatment, the expression levels of Foxn4 were significantly up-regulated in the L/C, M/C, and H/C groups. As a transcription factor, Myt1l is mainly transcribed in mouse embryonic brain and involved in neuron projection development [57,58] and neurogenesis [59,60]. Myt1l is also associated with nervousness [61], depressive disorder [62,63] and schizophrenia [64]. Stevens certificated that Myt1l can direct the conversion of human fibroblasts into functional neurons in combination with two other transcription factors, suggesting that Myt1l is significant in the development of cognitive functions [59]. On the basis of our results, the expression levels of Myt1l significantly increased in the L/C and H/C groups. Moreover, Kruer identified that homozygous mutations in the gene for fatty acid 2-hydroxylase (Fa2h) was implicated in abnormalities of ceramide metabolism in the pathogenesis of neurodegeneration with brain iron accumulation [65]. In accordance with our data, the expression of Fa2h was altered in the L/C group. Down syndrome cell adhesion molecules-like 1 (Dscaml1) is a paralogous protein of DSCAMs, a group of immunoglobulin-like transmembrane proteins [66]. Zhang showed that Dscaml1 knockdown increases the complexity of the proximal dendritic branch and inhibits the growth of axons in cultured neurons. Zhang also found that Dscaml1 promotes normal radial migration and callosal projection in in vivo knockdown experiments during postnatal development [67]. In the present study, the expression of Dscaml1 significantly changed in the L/C group. Wollmer showed that Hmgcs2, a protein related to cholesterol metabolism, is associated with AD at a significance level of p ≤ 0.05 and contributes to AD risk [68]. In the present data, the expression of Hmgcs2 significantly changed in the H/C group. In addition to the aberrantly expressed mRNAs, a number of lncRNAs were detected and differentially expressed. To the best of our knowledge, the potential functions and related diseases of most lncRNAs remain unknown to date. However, Tables 1, 2, 3, 4, 5 and 6 show that lncRNA BC079195 was consistently up-regulated while lncRNAs uc.229- and BC089928 were consistently down-regulated in the L/C, M/C, and H/C groups. This result suggests that these lncRNAs participate in the pathogenesis of Mn-induced diseases in the central nervous system. Further research is warranted explore the related functions of these lncRNAs in human diseases. The results of differentially expressed lncRNAs/mRNAs may provide evidence on the mechanisms underlying Mn-induced neurotoxic effects. Moreover, aberrantly expressed lncRNAs/mRNAs may be useful in further research of indicating early symptoms of Mn-induced neurotoxicity.

To reveal the functional significance of aberrantly expressed mRNAs in Mn-induced neurotoxicity, we constructed GO and pathway analysis. GO analysis demonstrated that aberrantly expressed mRNAs were associated with numerous specific biological processes (e.g., development), cellular components (e.g., synapse and cell periphery), molecular functions (e.g., protein binding ability). Pathway analysis indicated that differentially expressed mRNAs were also targeted to several specific signaling pathways, including GABAergic synapse (rno04727), glutamatergic synapse (rno04724), dopaminergic synapse (rno04728), synaptic vesicle cycle (rno04721), axon guidance (rno04360), apoptosis (rno04210), neurotrophin signaling pathway (rno04722), neuroactive ligand-receptor interaction (rno04080) and p53 signaling pathway (rno04115) (S5 Table). Of the aforementioned signaling pathways, available evidence in vivo experiments indicated that Mn-induced neurotoxicity may be mediated by dysregulation of gamma-aminobutyric acidergic (GABAergic), glutamatergic and dopaminergic neurotransmitter systems [69,70]. In our present study, the expression levels of Cacna1d, Gnb3, and Gng5 were all significantly altered in GABAergic synapse, glutamatergic synapse, and dopaminergic synapse (S3, S4, and S5 Figs), suggesting these aberrantly expressed transcripts may be highly associated with Mn-induced neurotoxicity. At the nerve terminal, synaptic vesicles cycle contains three pivotal processes: release of neurotransmitter from vesicle exocytosis, endocytosis of empty vesicles, and regeneration of fresh vesicles. Among these, vesicle exocytosis with neurotransmitter release takes participate in the communication between neurons [71]. On the basis of our results, the expression of Atp6vod2, Atp6v1b2, Cacna1b, Clta, Cplx1, Dnm1, Rab3a, Slc17a6, Slc18a1, Slc32a1, Stx1a, Stxbp1 significantly changed in synaptic vesicle cycle (S6 Fig). Neurons need to form an intricate network, and then function properly in biological organisms. Axon guidance represents a critical step in the formation of neuronal network and is regulated by a number of guidance molecules [72]. In our present study, the expression levels of L1cam, Lrrc4, Lrrc4c, Ngef, Pak3, Plxnb3, Robo1, Sema4c, Sema5a were also altered in axon guidance (S7 Fig). Apoptosis is generally recognized as the best-understood mechanism of neuronal cell death. Neuronal apoptosis is not only involved the developing brain, but also plays a potentially critical role in neurodegeneration [73]. Furthermore, neurotrophin signalling pathways have now been shown to regulate neuronal apoptosis through the action of pivotal protein kinase cascades [74]. For neuroactive ligand-receptor interaction, an available study demonstrated that this pathway was identified by extended Bayesian lasso (EBLasso) when applying to a genome-wide association study (GWAS) dataset for Parkinson disease [75]. Kong conducted a Drosophila Parkinson's disease model and reported that the dysregulated miRNAs were target to neuroactive ligand-receptor interaction pathway in vivo [76]. These signaling pathways are tightly implicated in the pathological processes, especially in neurodegenerative diseases. According to our research, a variety of genes were differentially expressed in these pathways (S8, S9, and S10 Figs), suggesting the dysregulated mRNAs play a vital role in the pathogenesis of Mn-induced neurotoxicity. These findings may also provide novel insights into potential mechanisms involved in Mn-induced neurological dysfunction. For instance, p53 is universally regarded as a major regulator of stress responses, and p53 pathway is rapidly activated by a number of stress signals, including DNA damage, hypoxia, oxidative stress and activated oncogenes. Wan demonstrated that by means of both transcription-dependent and -independent mechanisms, p53 was critically involved in Mn-induced neuronal apoptosis in rat striatum, and further illustrated the vital role of p53 pathway in the pathogenesis of manganism [77]. This result was consistent with ours, indicating that some mRNAs were also significantly altered in p53 signaling pathway, such as Atm, Chek1 and Perp (S11 Fig).

In qRT-PCR validation, on the basis of the features of these differentially expressed lncRNAs, we selected three interesting candidate lncRNAs (UC.105-, MRuc009dte, and BC090328). Obg-like ATPase (OLA1) is the associated gene of UC.105-. Ola1 is reportedly involved in regulating cellular antioxidant response and functions as an antioxidant suppressor of the oxidative stress response. OLA1 overexpression prevents the damaging reactive oxygen species (ROS) from scavenging in cells [78,79]. Neurons are vulnerable to various stresses that can induce apoptosis, and neuronal death caused by oxidative stress is implicated in all neurodegenerative disorders. Croze indicated that OLA1 down-regulation is a plausible mediator of neuronal preservation, which was implicated in optic neuropathy and multiple sclerosis-like lesions [80]. ING4 is the associated gene of MRuc009dte, and its sustained expression facilitates apoptosis [81,82]. HOMER3 is the associated gene of BC090328 that specifically interacts with amyloid precursor protein. This gene is reportedly involved in the pathology of AD [83,84]. With regard to aberrantly expressed mRNAs, we also verified four related mRNAs (Caspase 4, Picalm, Foxo3, and Pde8a), which we thought may be associated with the pathogenesis of Mn-induced neurotoxicity in accordance with the following reasons: Caspase 4 can act as an endoplasmic reticulum (ER) stress-specific caspase and may be associated with the pathology of AD in human [85]. Furthermore, Wang performed a Mn-exposed model in adult SD rats and confirmed that ER stress and ER stress-mediated apoptosis were involved in Mn-induced neurotoxicity [86]. Picalm is an important gene that encodes phosphatidylinositol-binding clathrin assembly protein [87]. Several studies cited this gene as a risk factor for AD [88,89]. FOXO3, a member of the FoxO subfamily of forkhead transcription factors, functions in the regulation of apoptosis and in the defense against oxidative stress [90,91]. Hagenbuchner demonstrated that FOXO3 promotes excessive ROS production in neurons and subsequently triggers apoptosis [92]. The cyclic adenosine monophosphate (cAMP)-mediated second messenger signal pathway has is involved in learning and memory [93]. Meanwhile, Pde8a is a member of the cAMP selective phosphodiesterase family that specifically hydrolyzes cAMP [94]. Basing from these reports, we selected lncRNAs (UC.105-, MRuc009dte, and BC090328) and mRNAs (Caspase 4, Picalm, Foxo3, and Pde8a) for qRT-PCR validation. The qRT-PCR results were consistent with the microarray data, except for Picalm and Pde8a in the L/C group, and the relative expression levels of the three lncRNAs and four mRNAs were significantly up-regulated in L/C, M/C, and H/C groups.

In this study, we investigated the gene expression profiles of normal and Mn-treated SD rat hippocampal neurons through lncRNA microarray. The expression patterns of lncRNAs and mRNAs in Mn-treated cells significantly differed from those in normal hippocampal neurons. This result implies that Mn serves a key function in the dysregulation of these lncRNAs and mRNAs. Furthermore, we performed GO and pathway analyses on aberrantly expressed mRNAs. The results of bioinformatic methods showed that aberrantly expressed mRNAs were involved in multiple specific biological processes (e.g., development), cellular components (e.g., synapse and cell periphery), molecular functions (e.g., protein binding ability), and potentially related pathways (e.g., GABAergic synapse, glutamatergic synapse, dopaminergic synapse, synaptic vesicle cycle, axon guidance, apoptosis, neurotrophin signaling pathway, neuroactive ligand-receptor interaction and p53 signaling pathway). Through intersection analysis, we screened the consistently up-regulated and down-regulated lncRNAs and mRNAs. This information may be used in further studies. Inevitably, our study also had some limitations. On the one hand, we should combine the differentially expressed lncRNAs with the aberrant expression mRNAs to further determine the potential function and associated pathways of these lncRNAs. On the other hand, we should conduct research involving animal experiments and population studies to verify these differentially expressed lncRNAs. Therefore, future research will mainly focus on these two aspects.

In conclusion, the expression patterns of lncRNAs and mRNAs were significantly altered in the Mn-treated hippocampal neurons. Aberrantly expressed mRNAs were found to participate in some specific biological processes and were potentially involved in related pathways that may contribute to the pathogenesis of Mn-induced neurotoxicity. To the best of our knowledge, this study is the first to determine the expression profiles of lncRNAs and mRNAs in Mn-treated SD rat hippocampal neurons. Our current research may shed light on the biological significance of aberrantly expressed lncRNAs/mRNAs in the pathology of Mn-induced neurotoxic effects. Moreover, differentially expressed lncRNAs/mRNAs may provide new evidence to understand the underlying mechanisms and might be useful in further investigations to detect early symptoms of Mn-induced disease in the central nervous system. Thus, on the basis of our current study, an intriguing orientation for further research has been proposed, and comprehensive studies in the future are expected.

## Supporting Information

S1 Fig Cell viability of different manganese-treated primary hippocampal neurons. (PDF)

S2 Fig Neuronal morphology of different manganese-treated groups. A: In the control group, hippocampal neurons were plump and bright, dendritic branching of neurons formed network in a high density. B: In the low-Mn exposed group, the number of hippocampal neurons appeared a slight reduction and a minority of cells became round. C: In the intermediate-Mn exposed group, the number of hippocampal neurons decreased obviously, cytoplasm of neurons appeared shrinkage and dendritic branching became shorter. D: In the high-Mn exposed group, the dendritic branching of neurons became shorter or even disappeared, the cytoplasm shrunk into round groups and a majority of neurons died.
(PDF)

S3 Fig GABAergic synapse (rno04727). Yellow marked nodes are associated with down-regulated genes, orange marked nodes are associated with up-regulated genes, green nodes have no significance.
(PDF)

S4 Fig Glutamatergic synapse (rno04724). Yellow marked nodes are associated with down-regulated genes, orange marked nodes are associated with up-regulated genes, green nodes have no significance.
(PDF)

S5 Fig Dopaminergic synapse (rno04728). Yellow marked nodes are associated with down-regulated genes, orange marked nodes are associated with up-regulated genes, green nodes have no significance.
(PDF)

S6 Fig Synaptic vesicle cycle (rno04721). Yellow marked nodes are associated with down-regulated genes, orange marked nodes are associated with up-regulated genes, green nodes have no significance.
(PDF)

S7 Fig Axon guidance (rno04360). Yellow marked nodes are associated with down-regulated genes, orange marked nodes are associated with up-regulated genes, green nodes have no significance.
(PDF)

S8 Fig Apoptosis (rno04210). Yellow marked nodes are associated with down-regulated genes, orange marked nodes are associated with up-regulated genes, green nodes have no significance.
(PDF)

S9 Fig Neurotrophin signaling pathway (rno04722). Yellow marked nodes are associated with down-regulated genes, orange marked nodes are associated with up-regulated genes, green nodes have no significance.
(PDF)

S10 Fig Neuroactive ligand-receptor interaction (rno04080). Yellow marked nodes are associated with down-regulated genes, orange marked nodes are associated with up-regulated genes, green nodes have no significance.
(PDF)

S11 Fig P53 signaling pathway (rno04115). Yellow marked nodes are associated with down-regulated genes, orange marked nodes are associated with up-regulated genes, green nodes have no significance.
(PDF)

S1 Table Survival of different manganese-treated primary hippocampal neurons. (PDF)

S2 Table Apoptosis rate of manganese exposed neurons. (PDF)

S3 Table Primer information of 3 lncRNAs used for RT-qPCR analysis. (PDF)

S4 Table Primer information of 4 mRNAs used for RT-qPCR analysis. (PDF)

S5 Table The information of specific pathways. (XLS)
