# Supplementary material for: Expression Profiles of Long Noncoding RNAs and Messenger RNAs in Mn-Exposed Hippocampal Neurons of Sprague–Dawley Rats Ascertained by Microarray: Implications for Mn-Induced Neurotoxicity
Source: PLoS One. 2016 Jan 8;11(1):e0145856. doi: 10.1371/journal.pone.0145856 (PMC4706437; doi:10.1371/journal.pone.0145856)
Supplement: S1 Fig — (PDF) [file pone.0145856.s001.pdf]

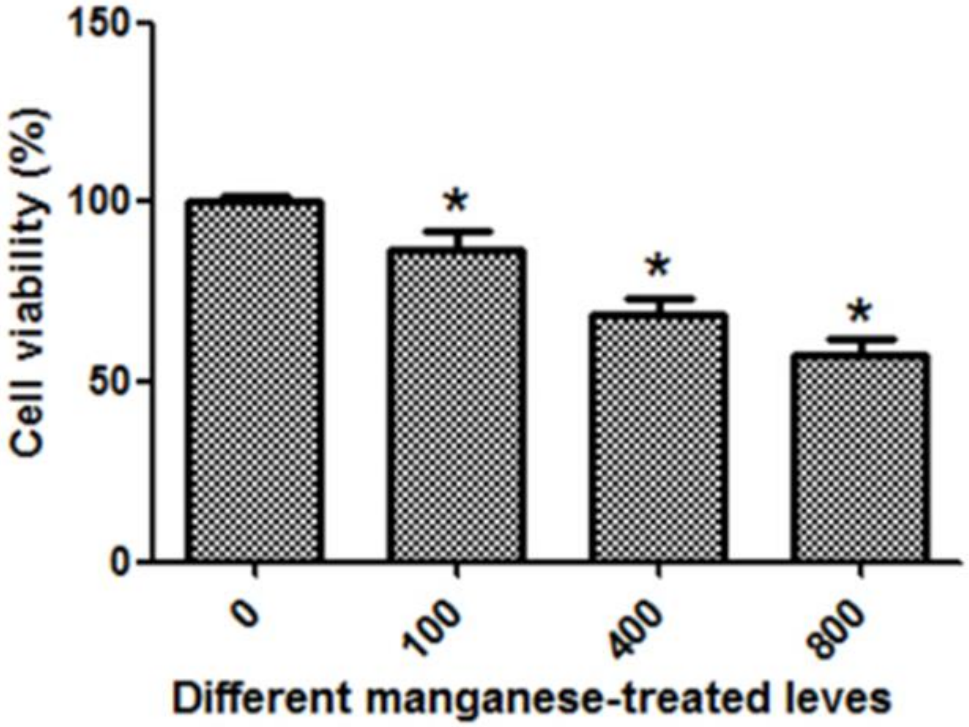

S1 Fig. Cell viability of different manganese-treated primary hippocampal neurons.

***: P< 0.05 when compared with the control group.**
