# Supplementary material for: Expression Profiles of Long Noncoding RNAs and Messenger RNAs in Mn-Exposed Hippocampal Neurons of Sprague–Dawley Rats Ascertained by Microarray: Implications for Mn-Induced Neurotoxicity
Source: PLoS One. 2016 Jan 8;11(1):e0145856. doi: 10.1371/journal.pone.0145856 (PMC4706437; doi:10.1371/journal.pone.0145856)
Supplement: S2 Fig — A: In the control group, hippocampal neurons were plump and bright, dendritic branching of neurons formed network in a high density. B: In the low-Mn exposed group, the number of hippocampal neurons appeared a slight reduction and a minority of cells became round. C: In the intermediate-Mn exposed group, the number of hippocampal neurons decreased obviously, cytoplasm of neurons appeared shrinkage and dendritic branching became shorter. D: In the high-Mn exposed group, the dendritic branching of neurons became shorter or even disappeared, the cytoplasm shrunk into round groups and a majority of neurons died. (PDF) [file pone.0145856.s002.pdf]

A.

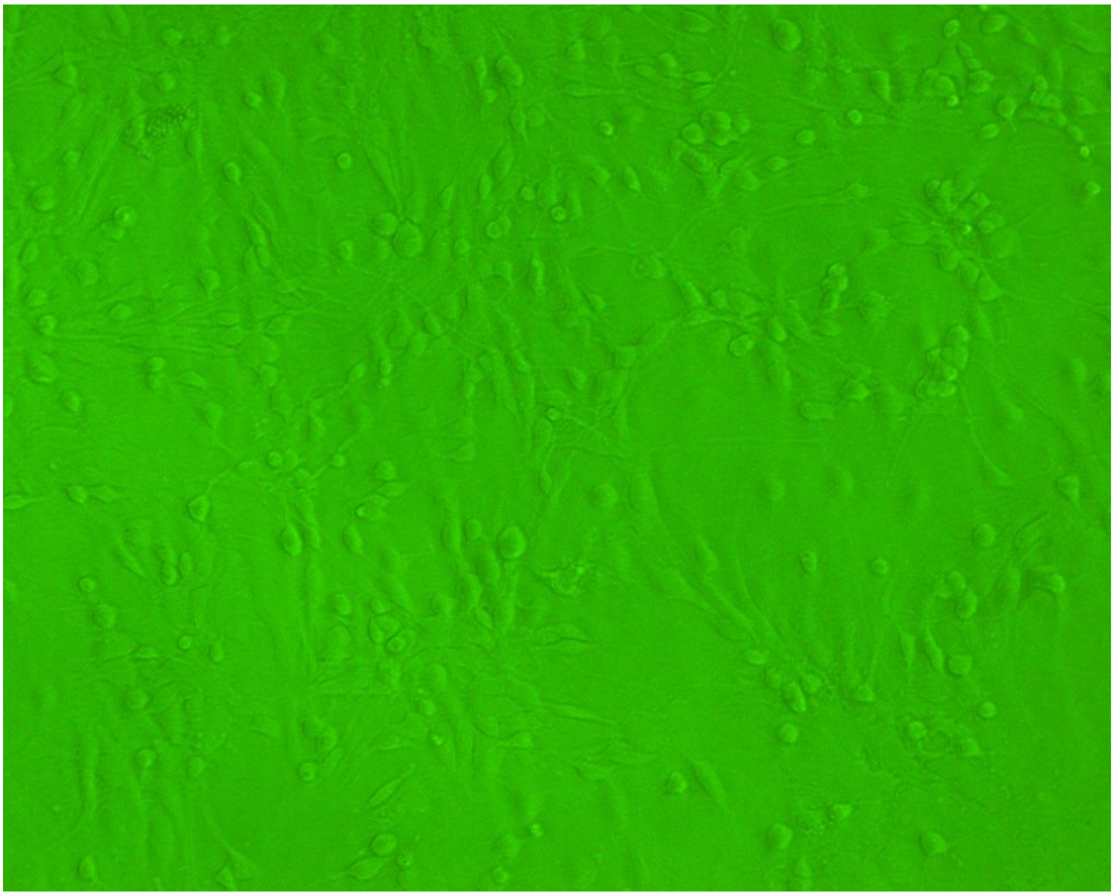

B.

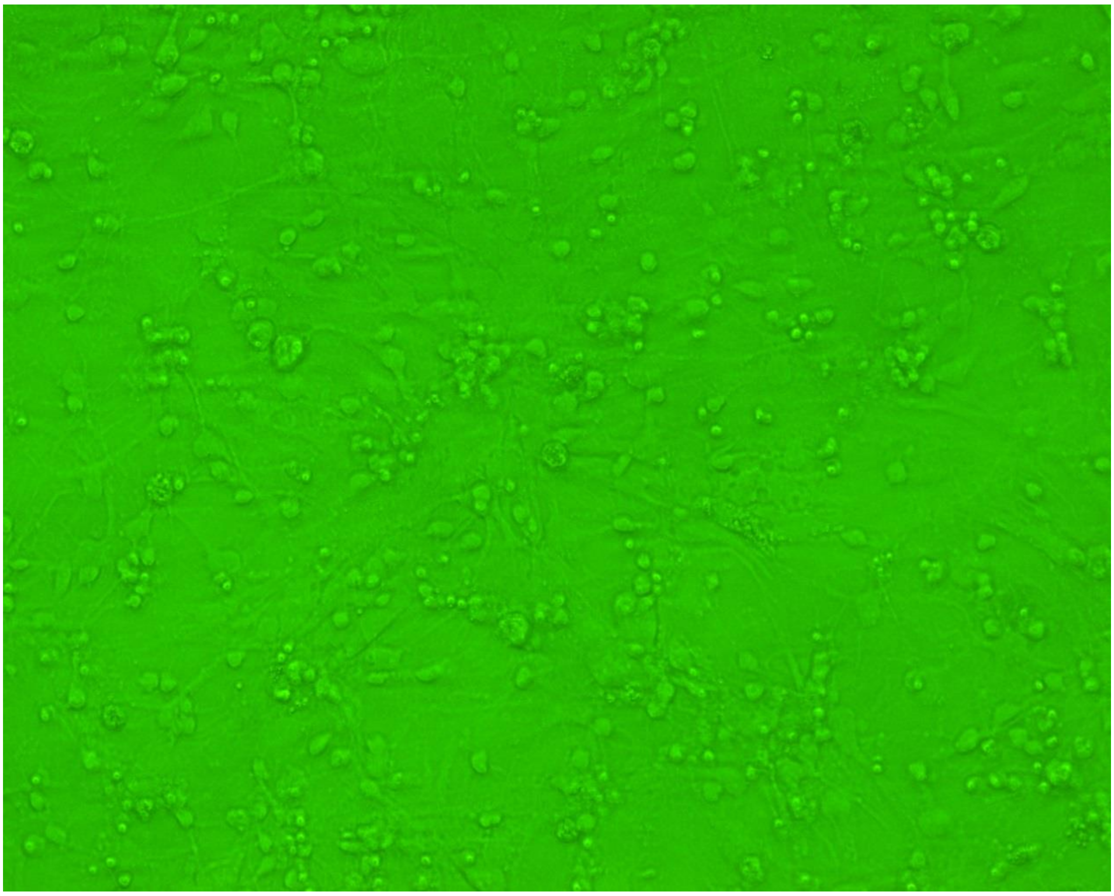

C.

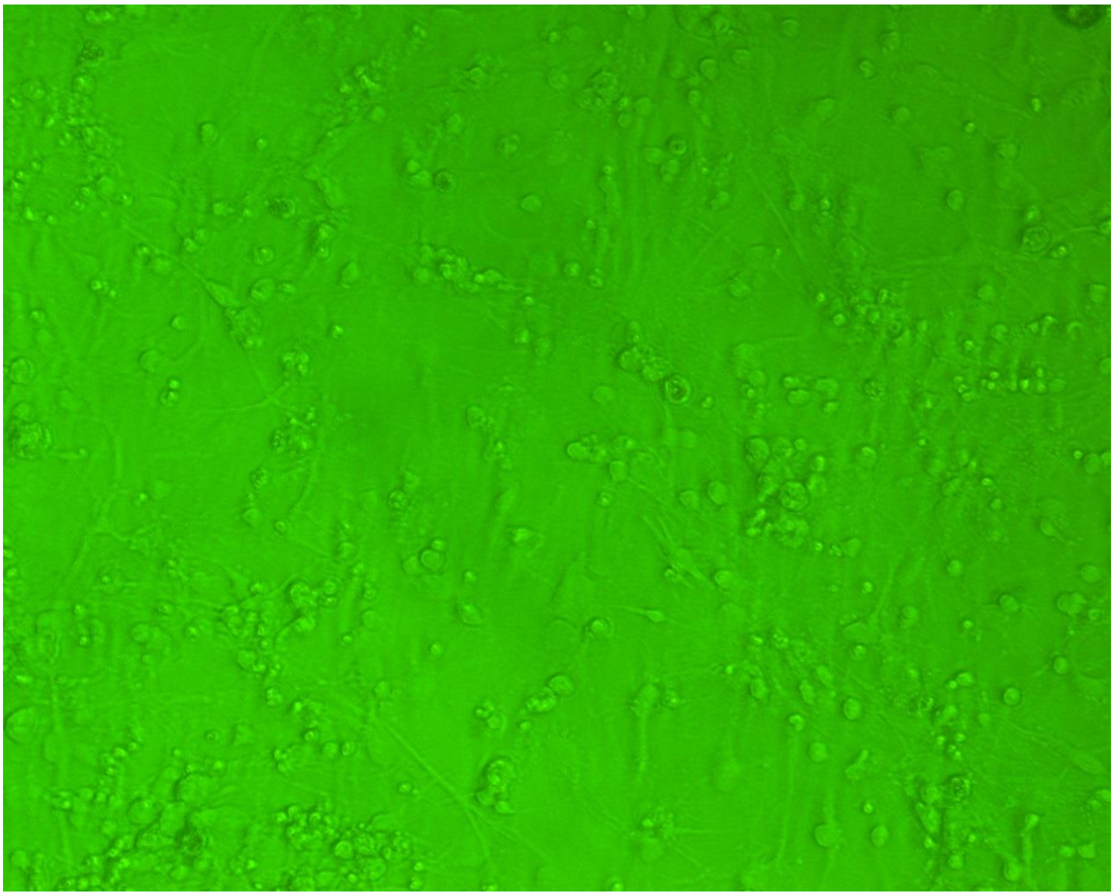

D.

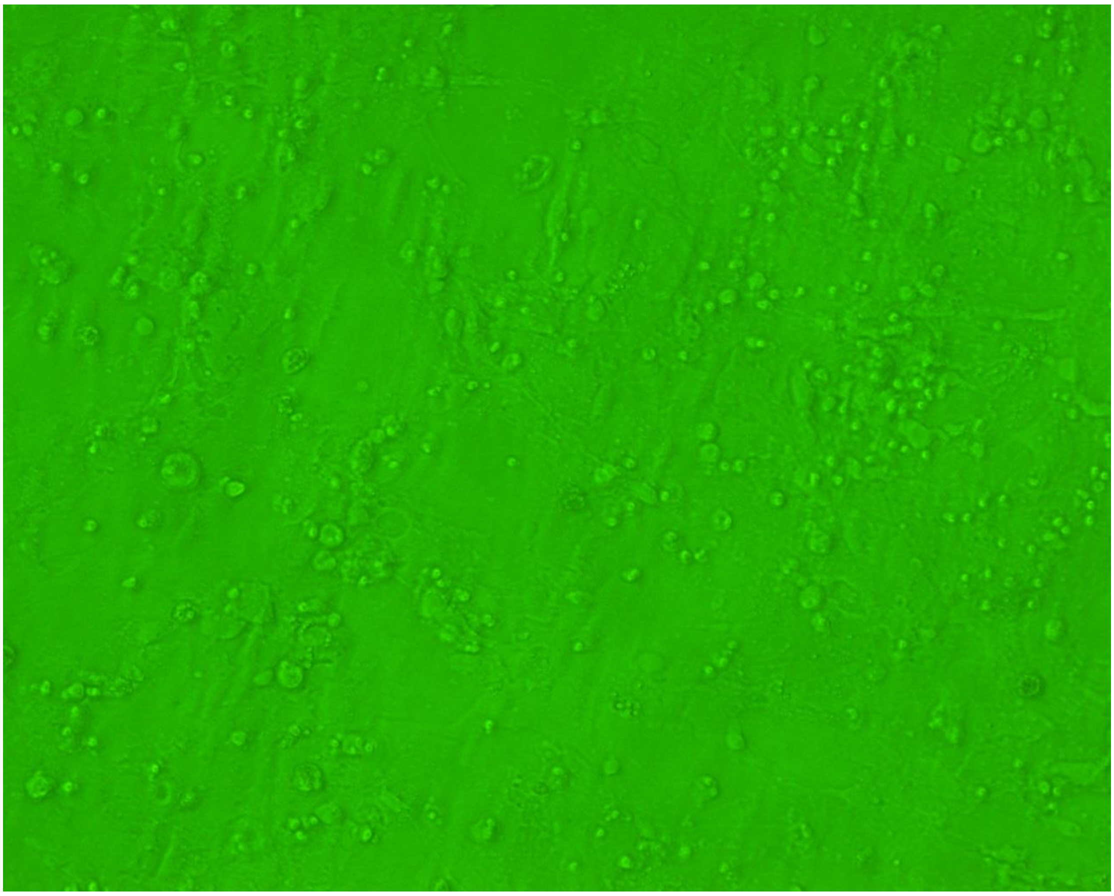

S2 Fig. Neuronal morphology of different manganese-treated groups. A: In the control group, hippocampal neurons were plump and bright, dendritic branching of neurons formed network in a high density. B: In the low-Mn exposed group, the number of hippocampal neurons appeared a slight reduction and a minority of cells became round. C: In the intermediate-Mn exposed group, the number of hippocampal neurons decreased obviously, cytoplasm of neurons appeared shrinkage and dendritic branching became shorter. D：In the high-Mn exposed group, the dendritic branching of neurons became shorter or even disappeared, the cytoplasm shrunk into round groups and a majority of neurons died.
