# Supplementary figures and images for: Expression Profiles of Long Noncoding RNAs and Messenger RNAs in Mn-Exposed Hippocampal Neurons of Sprague–Dawley Rats Ascertained by Microarray: Implications for Mn-Induced Neurotoxicity
Source: PLoS One. 2016 Jan 8;11(1):e0145856. doi: 10.1371/journal.pone.0145856 (PMC4706437; doi:10.1371/journal.pone.0145856)

# GABAergic SYNAPSE

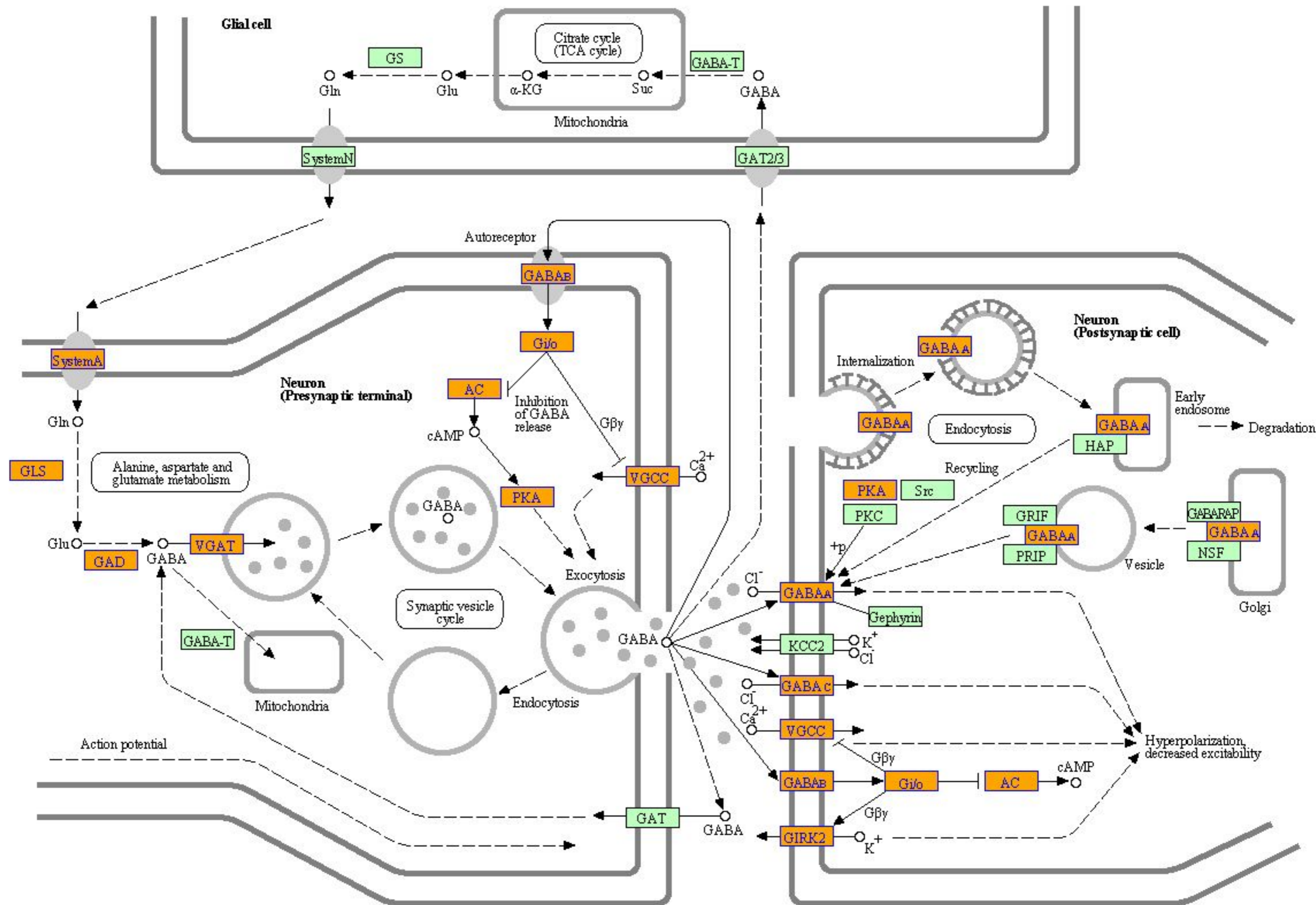

Supplement: S3 Fig — Yellow marked nodes are associated with down-regulated genes, orange marked nodes are associated with up-regulated genes, green nodes have no significance. (PDF) [file pone.0145856.s003.pdf]

# GLUTAMATERGIC SYNAPSE

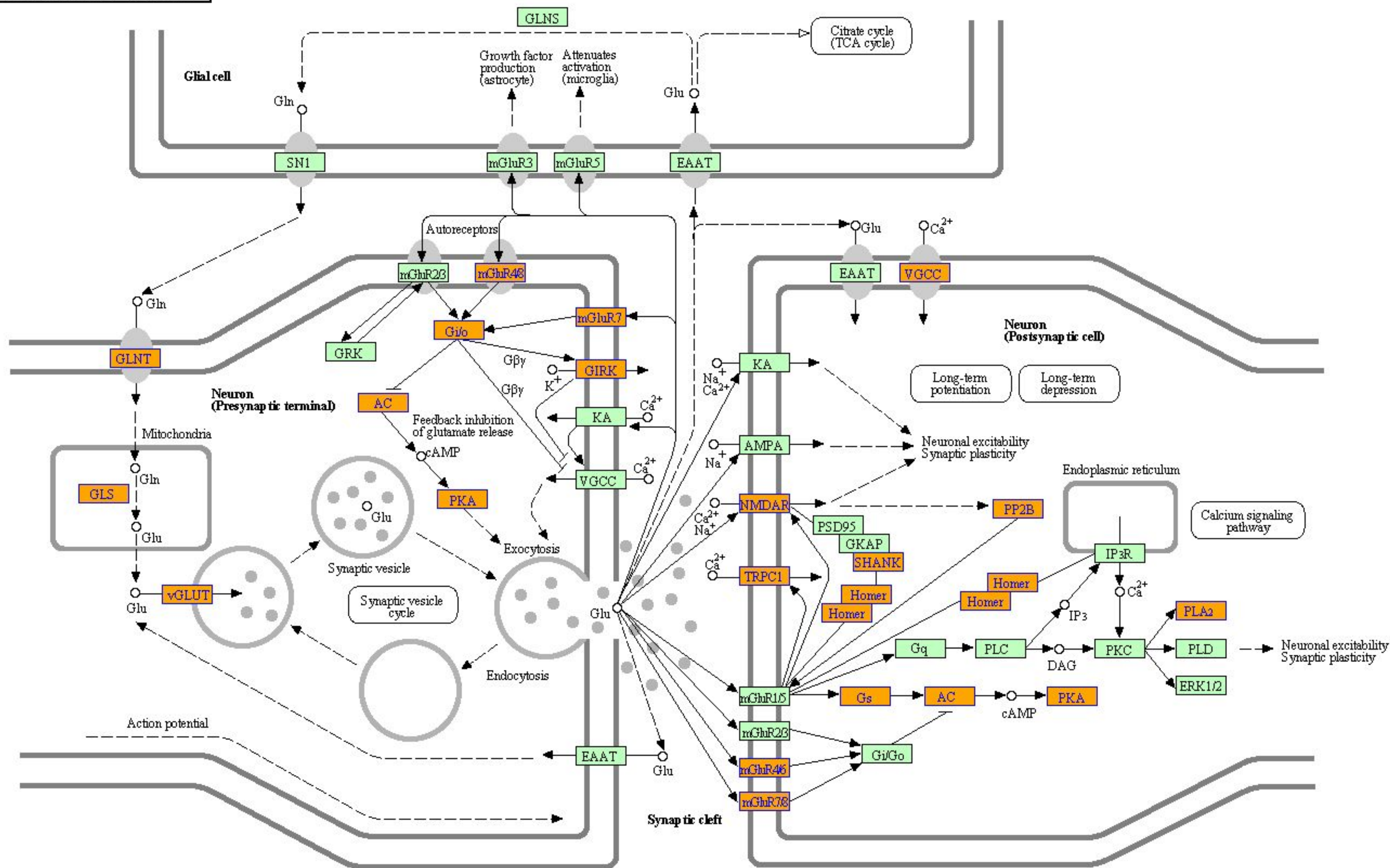

Supplement: S4 Fig — Yellow marked nodes are associated with down-regulated genes, orange marked nodes are associated with up-regulated genes, green nodes have no significance. (PDF) [file pone.0145856.s004.pdf]

# DOPAMINERGIC SYNAPSE

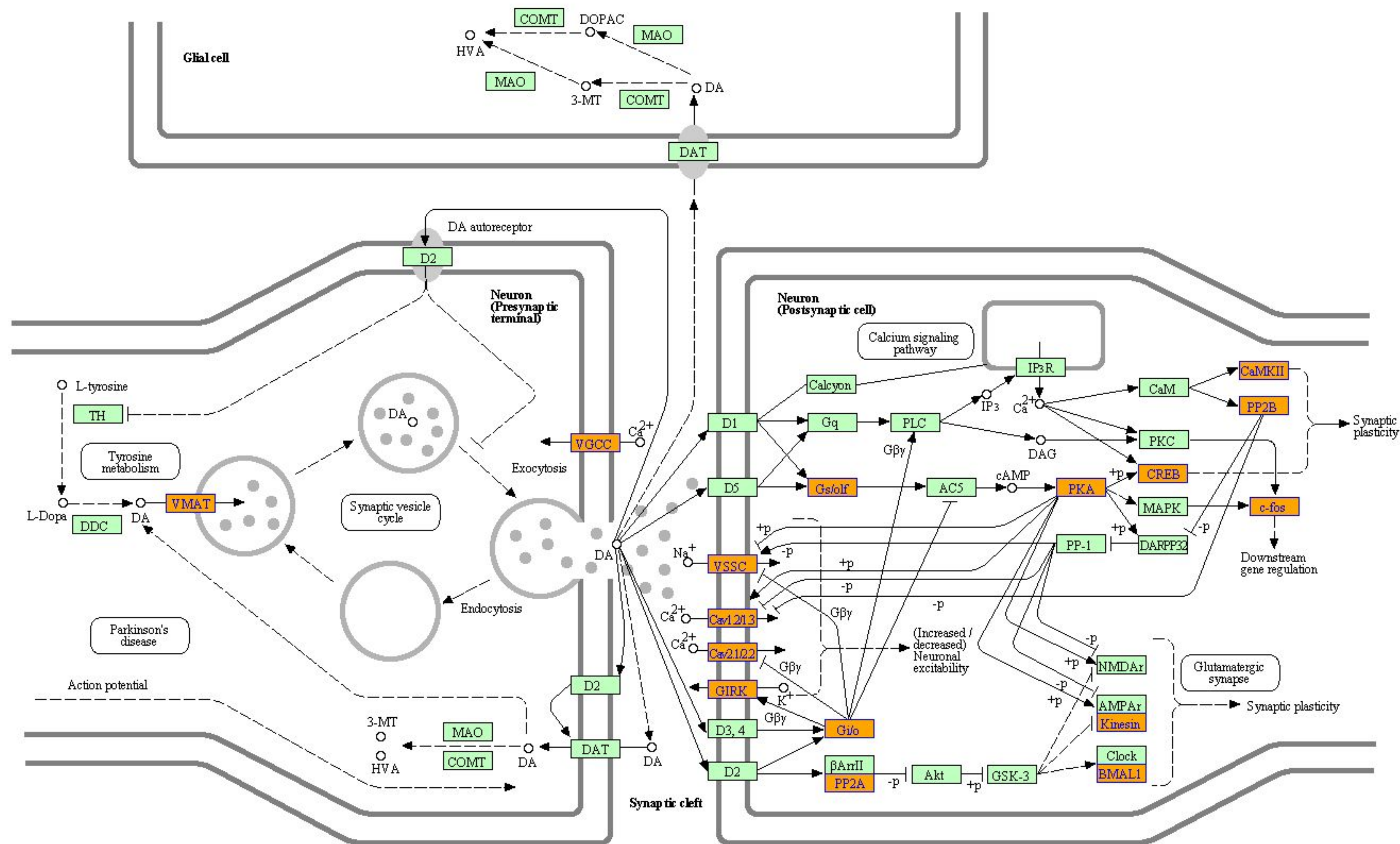

Supplement: S5 Fig — Yellow marked nodes are associated with down-regulated genes, orange marked nodes are associated with up-regulated genes, green nodes have no significance. (PDF) [file pone.0145856.s005.pdf]

SYNAPTIC VESICLE CYCLE

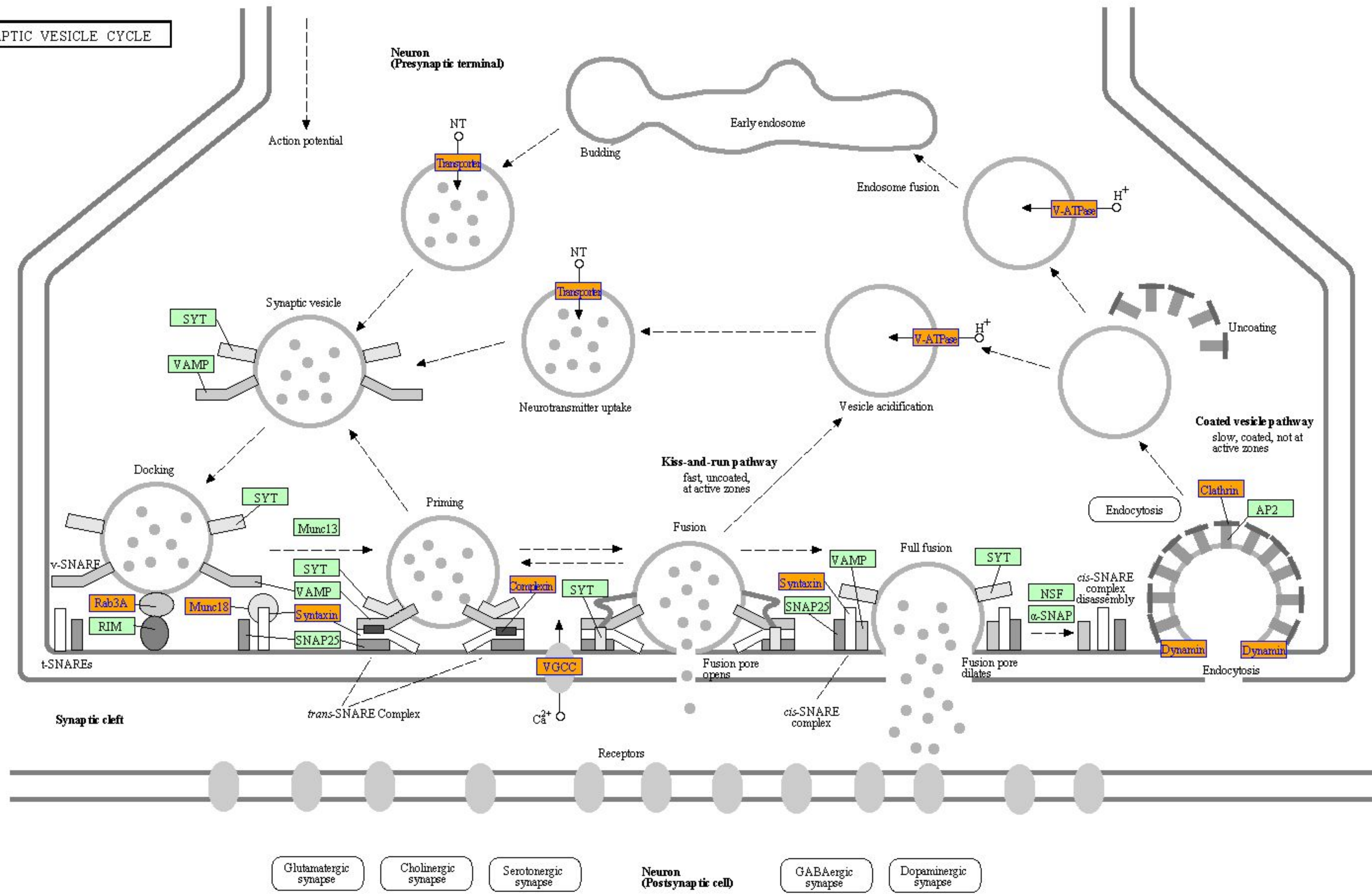

Supplement: S6 Fig — Yellow marked nodes are associated with down-regulated genes, orange marked nodes are associated with up-regulated genes, green nodes have no significance. (PDF) [file pone.0145856.s006.pdf]

# AXON GUIDANCE

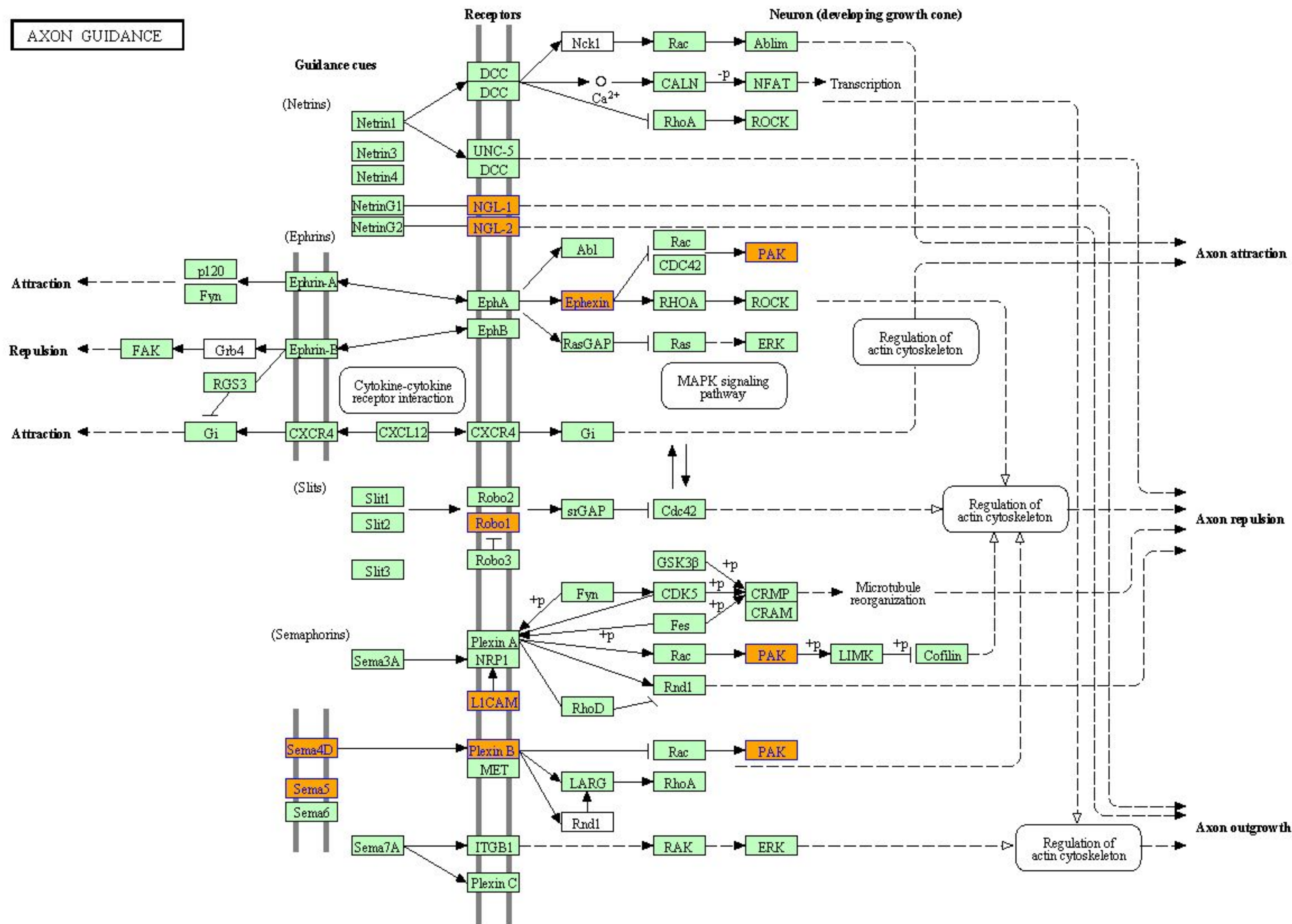

Supplement: S7 Fig — Yellow marked nodes are associated with down-regulated genes, orange marked nodes are associated with up-regulated genes, green nodes have no significance. (PDF) [file pone.0145856.s007.pdf]

# NEUROTROPHIN SIGNALING PATHWAY

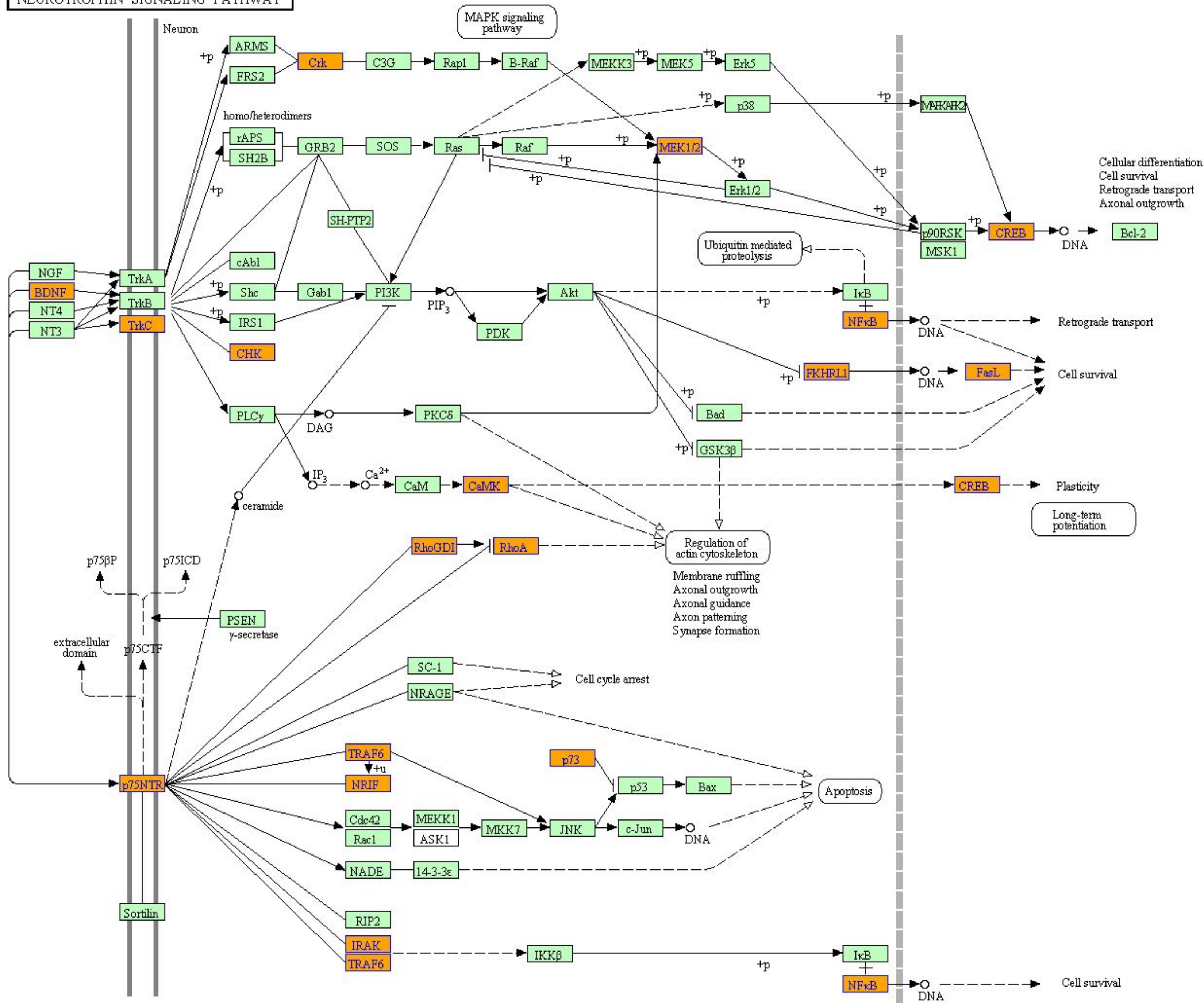

Supplement: S9 Fig — Yellow marked nodes are associated with down-regulated genes, orange marked nodes are associated with up-regulated genes, green nodes have no significance. (PDF) [file pone.0145856.s009.pdf]

# P53 SIGNALING PATHWAY

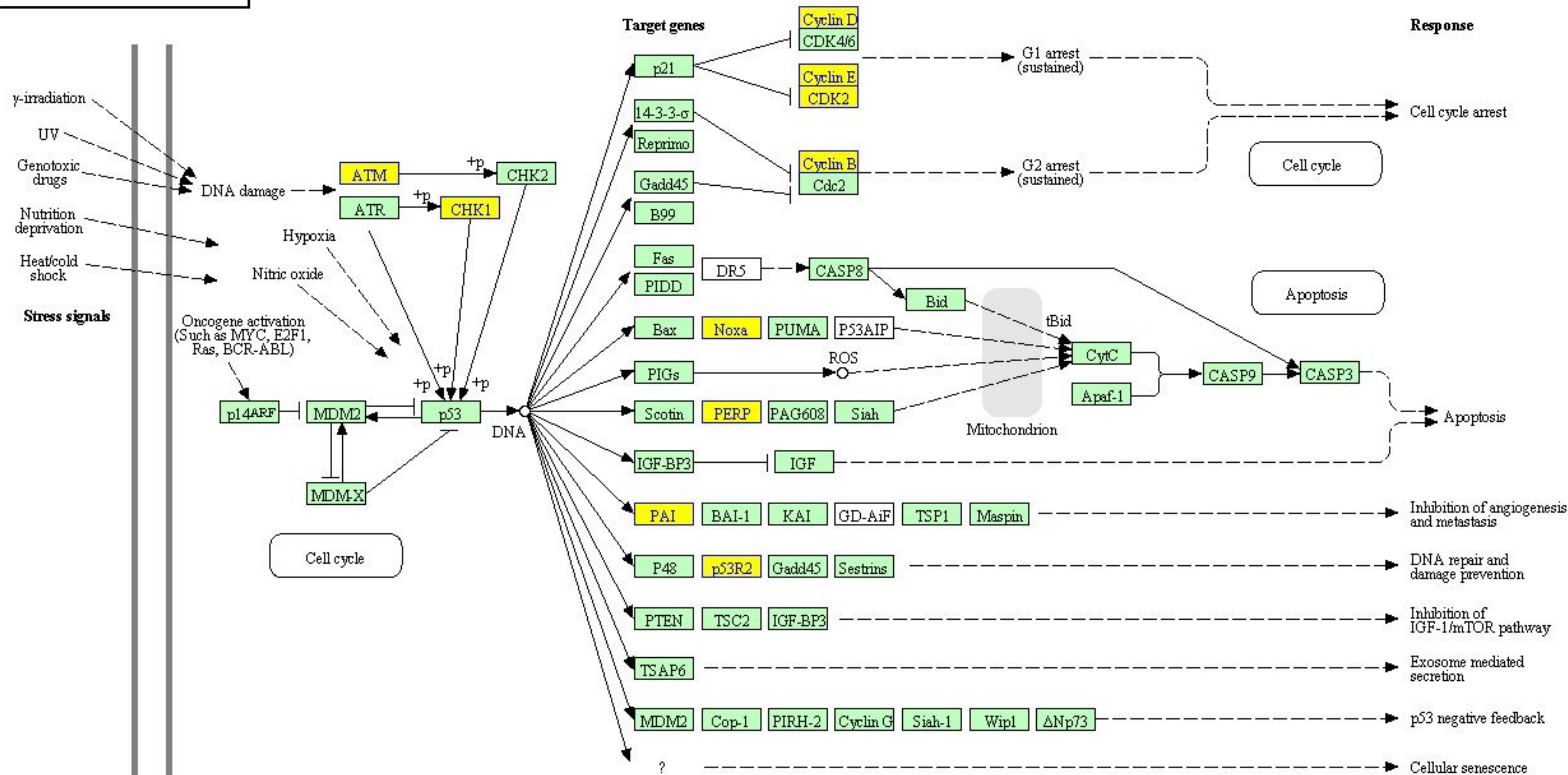

Supplement: S11 Fig — Yellow marked nodes are associated with down-regulated genes, orange marked nodes are associated with up-regulated genes, green nodes have no significance. (PDF) [file pone.0145856.s011.pdf]
