# Supplementary material for: Expression Profiles of Long Noncoding RNAs and Messenger RNAs in Mn-Exposed Hippocampal Neurons of Sprague–Dawley Rats Ascertained by Microarray: Implications for Mn-Induced Neurotoxicity
Source: PLoS One. 2016 Jan 8;11(1):e0145856. doi: 10.1371/journal.pone.0145856 (PMC4706437; doi:10.1371/journal.pone.0145856)
Supplement: S10 Fig — Yellow marked nodes are associated with down-regulated genes, orange marked nodes are associated with up-regulated genes, green nodes have no significance. (PDF) [file pone.0145856.s010.pdf]

NEUROACTIVE LIGAND-RECEPTOR INTERACTION

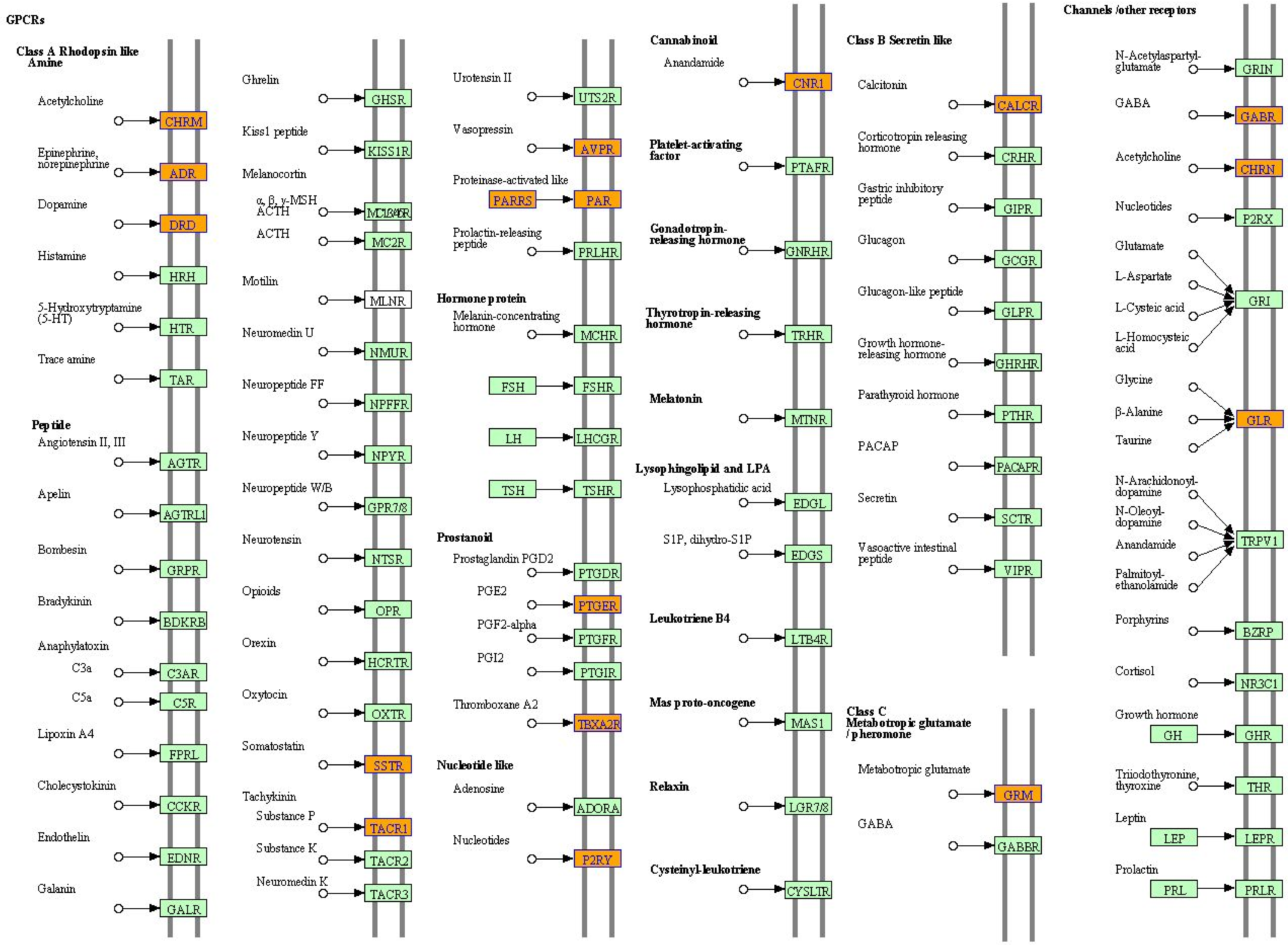

04080 8/30/11
(c) Kanehisa Laboratories
