# Supplementary material for: Expression Profiles of Long Noncoding RNAs and Messenger RNAs in Mn-Exposed Hippocampal Neurons of Sprague–Dawley Rats Ascertained by Microarray: Implications for Mn-Induced Neurotoxicity
Source: PLoS One. 2016 Jan 8;11(1):e0145856. doi: 10.1371/journal.pone.0145856 (PMC4706437; doi:10.1371/journal.pone.0145856)
Supplement: S1 Table — (PDF) [file pone.0145856.s012.pdf]

S1 Table. Survival of different manganese-treated primary hippocampal neurons ($\bar{X}$±SD, %)

**Mn-exposed（μmol/L）**	**0**	**100**	**400**	**800**
**Cell viability（100％）**	100±1.54	87.0±4.9*	68.9±4.4*	57.4±4.8*

*: Compared with the control group, $P < 0.05$.
