# Supplementary material for: Expression Profiles of Long Noncoding RNAs and Messenger RNAs in Mn-Exposed Hippocampal Neurons of Sprague–Dawley Rats Ascertained by Microarray: Implications for Mn-Induced Neurotoxicity
Source: PLoS One. 2016 Jan 8;11(1):e0145856. doi: 10.1371/journal.pone.0145856 (PMC4706437; doi:10.1371/journal.pone.0145856)
Supplement: S2 Table — (PDF) [file pone.0145856.s013.pdf]

S2 Table. Apoptosis rate of manganese exposed neurons ($\bar{X}$ ±SD, %)

Different manganese-exposed groups	Early apoptosis rate (%)	Late apoptosis rate (%)	Total apoptosis rate (%)
**Control group**	8.90 ±0.61	6.40 ±0.47	15.30 ±0.58
**Low Mn-exposed group**	9.10 ±0.15	7.70 ±1.60	16.82 ±1.55
**Intermediate Mn-exposed group**	17.60 ±3.22^{ab}	6.90 ±2.13	24.57 ±1.72^{ab}
**High Mn-exposed group**	20.10 ±9.01^{abc}	14.50 ±9.91^{abc}	34.70 ±1.15^{abc}

a: Compared with the control group, P< 0.05;

b: Compared with the low Mn-exposed group, *P*< 0.05;

c: Compared with the intermediate Mn-exposed group, *P*< 0.05.
