# Supplementary material for: Expression Profiles of Long Noncoding RNAs and Messenger RNAs in Mn-Exposed Hippocampal Neurons of Sprague–Dawley Rats Ascertained by Microarray: Implications for Mn-Induced Neurotoxicity
Source: PLoS One. 2016 Jan 8;11(1):e0145856. doi: 10.1371/journal.pone.0145856 (PMC4706437; doi:10.1371/journal.pone.0145856)
Supplement: S3 Table — (PDF) [file pone.0145856.s014.pdf]

S3 Table. Primer information of 3 lncRNAs used for RT-qPCR analysis.

Gene name	Bidirectional primer sequence	Annealing temperature(°C)	Length (bp)
**GAPDH**	F:5'GGAAAGCTGTGGCGTGAT3'<br>R:5'AAGGTGGAAGAATGGGAGTT3'	60	308
**UC.105-**	F:5'TGACGCACATAATTGCCTACA3'<br>R:5'GCAGCCTTGCTATTAACTGTTC3'	60	138
**MRuc009dte**	F:5'ACGAAGCCCACCGTCTGTA3'<br>R:5'AACTCCTCTGAGCCGCACC3'	60	169
**BC090328**	F:5'GAGGTCCCGTTTCTTGCTG3'<br>R:5'TGGCTGGTTGCTTCTTTCTC3'	60	179
