# Supplementary material for: Expression Profiles of Long Noncoding RNAs and Messenger RNAs in Mn-Exposed Hippocampal Neurons of Sprague–Dawley Rats Ascertained by Microarray: Implications for Mn-Induced Neurotoxicity
Source: PLoS One. 2016 Jan 8;11(1):e0145856. doi: 10.1371/journal.pone.0145856 (PMC4706437; doi:10.1371/journal.pone.0145856)
Supplement: S4 Table — (PDF) [file pone.0145856.s015.pdf]

S4 Table. Primer information of 4 mRNAs used for RT-qPCR analysis.

Gene name	Bidirectional primer sequence	Annealing temperature(°C)	Length (bp)
**GAPDH**	F:5'GGAAAGCTGTGGCGTGAT3'<br>R:5'AAGGTGGAAGAATGGGAGTT3'	60	308
**Caspase 4**	F:5'CAACACCACACCACTTGTCCTA3'<br>R:5'CTTTCCTCAGTTTCCCTAATCC3'	60	301
**Picalm**	F:5'TATCCCACAGTTGGCAGACAG3'<br>R:5'CGCTCGTTTCCATACACCATC3'	60	111
**Foxo3**	F:5'TTCCGTAAGCAAGCCGTGTA3'<br>R:5'AGTGACGCAGGTCCGAACA3'	60	252
**Pde8a**	F:5'CTGATGGCTTACGGAGGTTT3'<br>R:5'ATGAAGGGAGATGGGGACG3'	60	98
